# Structure variations within *R*Si_2_ and *R*_2_Si_3_ silicides. Part II. Structure driving factors

**DOI:** 10.1107/S2052520620003893

**Published:** 2020-05-15

**Authors:** M. Nentwich, M. Zschornak, M. Sonntag, R. Gumeniuk, S. Gemming, T. Leisegang, D. C. Meyer

**Affiliations:** a Institute for Experimental Physics,Technical University Bergakademie Freiberg, 09596 Freiberg, Germany; b Institute of Ion Beam Physics and Materials Research, Helmholtz-Zentrum Dresden-Rossendorf, 01328 Dresden, Germany; c Institute of Physics, Technische Universität Chemnitz, 09107 Chemnitz, Germany; d Samara Center for Theoretical Materials Science, Samara State Technical University, Samara, Russia

**Keywords:** silicide, rare earth, ordering phenomena, structure prediction, DFT

## Abstract

To gain an overview of the various structure reports on *R*Si_2_ and *R*_2_ *T*Si_3_ compounds (*R* is a member of the Sc group, an alkaline earth, lanthanide or actinide metal, *T* is a transition metal), compositions, lattice parameters *a* and *c*, ratios *c*/*a*, formula units per unit cell, and structure types are summarized in extensive tables and the variations of these properties when varying the *R* or *T* elements are analyzed. Following the structural systematization given in Part I, Part II focuses on revealing the driving factors for certain structure types, in particular, the electronic structure. Here, concepts of different complexity are presented, including molecular orbital theory, the principle of hard and soft acids and bases, and a Bader analysis based on Density Functional Theory calculations for representatives of the reported structure types. The potential Si/*T* ordering in different structures is discussed. Additionally, the influences from intrinsic and extrinsic properties (*e.g.* elemental size and electronics as well as lattice parameters and structure type) are investigated on each other using correlation plots. Thermal treatment is identified as an important factor for the ordering of Si/*T* atoms.

## Abstract 2

Most articles dealing with *R*Si_2_ and *R*_2_ *T*Si_3_ compounds are only interested in one specific compound or in a series of compounds with varying *T* elements while keeping *R* fixed (or vice versa). Here, the focus lies on the complete space of 2:1:3 and 1:2 silicides, discussing various crystallographic properties and reasons for the formation of the different symmetries and superstructures.

## 1. Introduction   

The response of a crystal structure to a change in composition depends on its ‘flexibility’ concerning varying atomic size and electronic structure (Hume-Rothery & Raynor, 1962 ▸). The crystal system responds with a change in atomic order or with atomic displacements (Leisegang *et al.*, 2005 ▸; Tang *et al.*, 2011 ▸; Nentwich *et al.*, 2014 ▸, 2016 ▸), and thus possibly with a change of the structure type. In the substitutional regime considered in the present work, the exchange of an element by another one is responsible for the modification of the composition.

The predictive power of modern electronic structure calculations has steadily become more reliable because of highly developed theories and available computational capacities. Nevertheless, the determination of slight structural deviations and pseudosymmetries as well as accompanied stabilities of certain structure types with respect to specific substitutional exchange reflects fundamental issues in the chemistry of intermetallic compounds. Especially the interpretation of chemical bonds is very complex and in many cases not completely understood. Therefore, information on the structure, stability, and physical properties of intermetallic compounds are important in order to develop a better comprehension of structural features such as element ordering and the respective driving forces.

In this regard, the rare earth compounds are highly attractive, as they exhibit very diverse properties from magnetism to superconductivity, in dependence on the rare earth element (Sc, Y, La, …, Lu), the crystal structure, and possibly transition metal substitutions, see Bertaut *et al.* (1965 ▸) and Wunderlich *et al.* (2010 ▸). In the past few decades, the rare earth disilicides *R*Si_2_ have become an object of numerous studies mainly due to their exciting magnetic properties, in particular upon substituting one in four Si atoms by a transition metal *T* (*R*_2_ *T*Si_3_ compounds)

**Figure 1:**
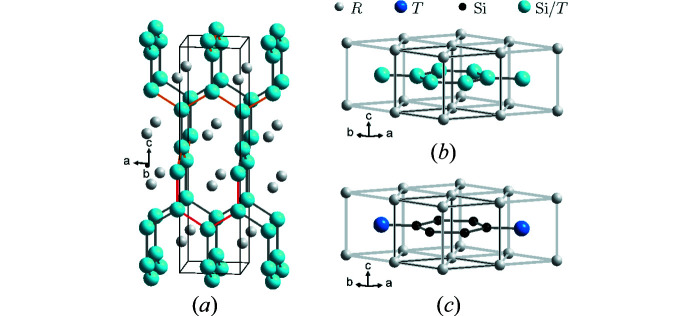
(*a*) Tetragonal ThSi_2_ and (*b*) hexagonal AlB_2_ structures of *R*Si_2_ and disordered as well as (*c*) ordered AlB_2_-like *R*_2_ *T*Si_3_ compounds (unit cell outline in black). The AlB_2_ structures form a 2D sublattice of hexagonally arranged Si atoms (red bonds). In contrast, the ThSi_2_ structures form 3D networks with incomplete hexagons (red bonds), accompanied with the formation of zigzag chains alternately in *a* and *b* directions *ab* direction along the *c* stacking (orange bonds).

These compounds can be divided according to two main classes of structure types: the AlB_2_- and the ThSi_2_-type, based on the hexagonal space group *P*6/*mmm* (No. 191) (Hofmann & Jäniche, 1935 ▸) and the tetragonal space group *I*4_1_/*amd*, (No. 141) (Brauer & Mittius, 1942 ▸), see Figs. 1 ▸(*a*) and 1 ▸(*b*). Both structure types also arise in compounds with actinide and alkaline earth metals of identical stoichiometry. Thus, we enhanced our database by these two groups.

We have been systematizing the large variety of structure types within the *R*Si_2_ and *R*_2_ *T*Si_3_ compounds in a Bärnig­hausen diagram in Part I of this work (Nentwich *et al.*, 2020 ▸). Here, we only distinguish between the two aristotypes and, additionally, their orthorhombic derivatives. We focus on the influence of structural and electronic parameters of both the *R* and the *T* element to reveal the structure driving factors. We employ parameters related to the complete compound such as lattice parameters, smallest *d* distances, and application of thermal treatment, to elemental size such as radii of the elements and their ratio, and to elemental electronics such as valence electrons.

We used different approaches to visualize potential relations between these parameters, *i.e.* boxplots, correlation plots, and *R*–*T* plots, which we already introduced in Part I (Nentwich *et al.*, 2020 ▸).

## 2. Methods   

### 2.1. Data collection   

We extracted the data for this work from over 300 articles presenting experimental structure reports at ambient conditions, without further refinement. However, we did not consider data sets if they were too incomplete, *i.e.* missing lattice parameters, non-ambient conditions or insufficient symmetry description. Additionally, we excluded structure reports of ternary compounds with stoichiometries other than *R*_2_ *T*Si_3_ from our standard screening. The complete table with structure parameters, such as lattice parameters *a* and *c*, ratios *c*/*a*, formula units per unit cell, and structure types are listed in Appendix A of Part I (Nentwich *et al.*, 2020 ▸). Please note that the same sample was sometimes used in different publications, which has been indicated accordingly.

### 2.2. Element specific data   

We used reference values of the elements (such as electron configuration, atomic radii and mass) from Holleman & Wiberg (2007 ▸), with some minor extensions from Riedel & Janiak (2011 ▸), and references therein.

### 2.3. DFT-calculated Bader analysis   

The Bader analysis presented here is based on DFT calculations from Part I (Nentwich *et al.*, 2020 ▸), which use the projector-augmented wave (PAW) method (Kresse & Joubert, 1999 ▸) in spin-polarized PBE parametrization (Perdew *et al.*, 1996 ▸) implemented in the VASP code (Kresse & Furthmüller, 1996 ▸). Among other values, we present the difference between calculated and nominal valence electron amount, determined by the respective PAW potential: Nd—14; Ni, Pd—10; Cu, Ag—11, Si—4.

### 2.4. Visualization   

#### 2.4.1. Clustering the compounds according to their *R* and *T* elements   

**Figure 2:**
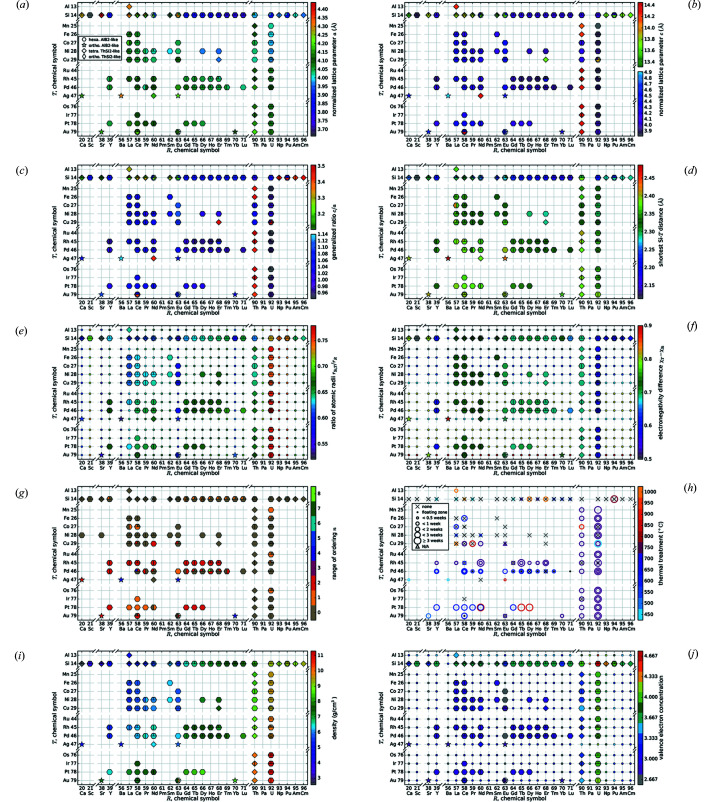
*R*–*T* diagram of the *R*Si_2_ and *R*_2_ *T*Si_3_ compounds. (*a*) normalized lattice parameter *a*, (*b*) normalized lattice parameter *c*, (*c*) normalized ratio *c*/*a* of lattice parameters, (*d*) shortest Si—*T* bonds, (*e*) ratio of atomi radii *r*_*T*,Si_/*r_R_*, (*f*) electronegativity difference EN(Si,*R*), (*g*) range of ordering, (*h*) thermal treatment, (*i*) density and (*j*) valence electron concentration. The used markers symbolize the crystal system: hexagon — hexagonal AlB_2_-like systems, open star — orthorhombic, AlB_2_-like systems, diamond — tetragonal ThSi_2_ systems, elongated diamond — orthorhombic GdSi_2_ systems. The lattice parameters *a* and *c* of the subplots (*a*) and (*b*) are normalized to the *R*–*R* distances within *a*/*b* and along *c*, respectively, to provide comparability. Accordingly, the ratio *c*/*a* in subplot (*c*) is also based on these normalizations. The shortest Si/*T* bonds in subplot (*d*) are calculated based on the formula (1). Subplot (*e*) depicts the ratio of atomic radii *q*_rad_, which is based on equation (2). Subplot (*f*) shows the electronegativity difference for the evaluation of the Zintl conditions. For the range of ordering *n* in subplot (*g*), a structure with disordered Si/*T* sites is marked with a black symbol, otherwise the color stands for the range of ordering *n*. For the thermal treatment (*h*), the color represents the temperature and the circle size the time of the treatment (triangle if unknown). Application of the floating zone method is marked with a black dot •, while no treatment is marked with a cross ×. The plots for the theoretical properties vec and ratio of radii were completed for not experimentally determined compounds (small circles) (Riedel & Janiak, 2011 ▸).

To compare the influence of substitution by an *R* or by a *T* element on a specific property, we adapted the *R*–*T* plot from Part I (see Fig. 2 ▸). These diagrams consist of a grid with the different *R* elements on the *x* axis and the *T* elements on the *y* axis, sorted by their atomic numbers.

The markers on the grid points generally symbolize the symmetry by shape (hexagonal AlB_2_-like: hexagon; orthorhombic AlB_2_-like: open star; tetragonal ThSi_2_: diamond; orthorhombic GdSi_2_: elongated diamond). The color visualizes the value of the parameter at the corresponding composition. For technical reasons, the *R*–*T* diagrams show at most three reports of the same compound. Our algorithm chooses the datasets with the highest as well as the lowest *a* parameter and an additional dataset with a different structure type to depict the most significant variations. The datasets from the complete list given in Appendix A of Nentwich *et al.* (2020 ▸) that have not been used are shaded in blue. For parameters basing on purely theoretical values as the ratio of radii, we complemented the values of compounds that were not yet reported by small circles to allow the estimation of trends.

#### 2.4.2. Box plots   

**Figure 3:**
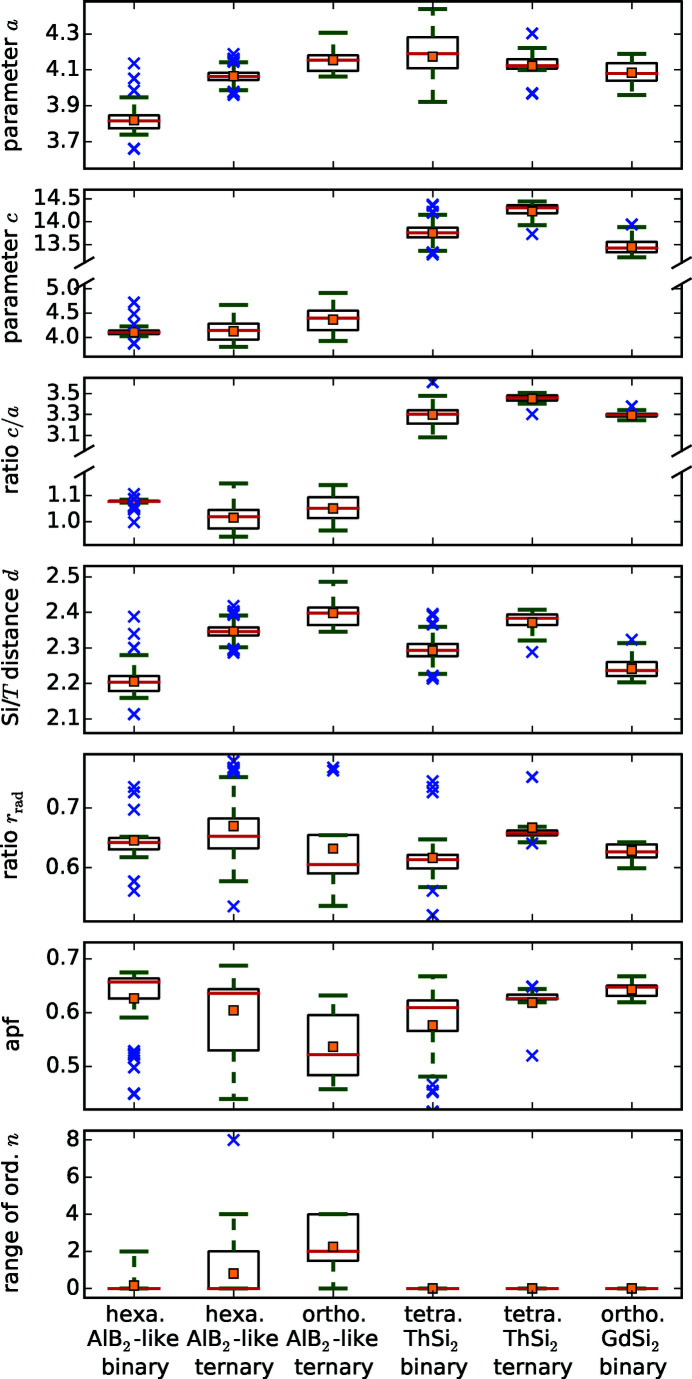
Box plots of the most important parameters, separated by the lattice and the composition of the compounds, if necessary. Orange square—average, red line—median, black box—limits of quartiles, green whiskers — 15th and 85th percentile, blue crosses — outliers.

The mathematical tool of box plots gives a first overview of the parameter variability in general, see Fig. 3 ▸. Box plots visualize various statistical parameters in one diagram: average (orange square), median (red line), quartiles (limits of black boxes), 15th/85th percentile (green whiskers), and outliers (blue cross). The median separates the lower from the higher half of a dataset. The quartiles separate the lowest 25% from the highest 75% and vice versa. The *x*th percentile separates the lowest *x*% from the highest 100 − *x*%. The box plots in Fig. 3 ▸ display the complete data range and split the data according to the lattice of the compounds and to the presence or absence of a *T* element. The latter is not necessary for the orthorhombic AlB_2_-like and orthorhombic GdSi_2_-type compounds as they only exist for ternary and binary compounds, respectively.

#### 2.4.3. Correlation plots   

**Figure 4:**
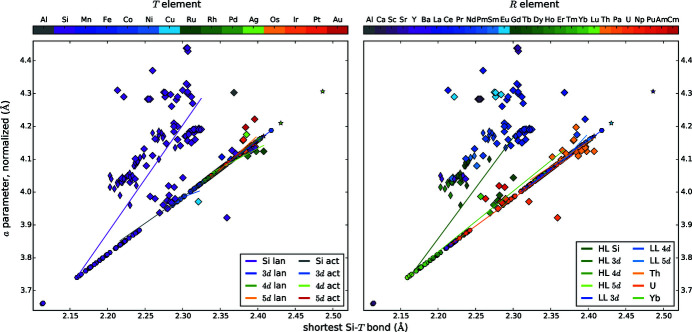
Correlations between the shortest Si—*T* distance *d* and the lattice parameter *a*. For AlB_2_-like compounds *a* ∝ *d* by definition, for ThSi_2_-like compounds the interrelationship is not linear due to distortions of the trigonal-planar coordination of Si/*T* atoms.

We present diagrams where two different parameters are plotted against each other to find correlations between them, see Figs. 4 ▸, 5 ▸, 6 ▸, 7 ▸, 8 ▸, 9 ▸, 10 ▸, 11 ▸ and 12 ▸. These diagrams hold manifold information. Every marker belongs to a dataset of the complete list given in Appendix A of Nentwich *et al.* (2020 ▸) and comprises its values for the two chosen parameters (position in *x* and *y* directions), its lattice type (shape) and its chemical composition (color). The symbols for the lattice types are the same as for the *R*–*T* diagrams: hexagon for hexagonal AlB_2_-like, open star for orthorhombic AlB_2_-like, diamond for tetragonal ThSi_2_ and elongated diamond for orthorhombic GdSi_2_. Each diagram consists of two versions of the same graph, to separately color mark the *T* and the *R* elements (left- and right-hand side of the figure, respectively). The *T* elements consist of the groups of 3d (blue), 4d (green), and 5d (orange/red) elements as well as Al (gray) and Si (purple). The *R* elements comprise light lanthanides (LL) [La, …, Gd; RÖMPP Online (2011 ▸)] (blue); heavy lanthanides (HL) [Tb, …, Lu; RÖMPP Online (2011 ▸)] (green) and actinides (orange/red) as well as alkaline earth metals and elements of the Sc group (gray/purple). We added lines to highlight the trend of certain subgroups, *e.g.* ‘4d lan’ means *R*_2_ *T*Si_3_ compounds with a lanthanide *R* and a 4d *T* element. However, in most cases the statistical interpretation of the slope is not reasonable as the corresponding data rather form clouds than lines.

## 3. Property overview (depending on *R* element, *T* element, and/or crystal symmetry)   

Two main factors influence the ability of an element to replace another one: the size and the electronic structure (Hume-Rothery & Raynor, 1962 ▸). Therefore, we chose the following groups of parameters for our study: (i) compound specific properties such as structure type, lattice parameters, shortest Si—*T* distance, atomic packing factor and *c*/*a* ratio as well as (ii) elemental size such as radius of the *R* and *T* element and ratio of elemental radii, and (iii) elemental electronic structure such as valence electrons and electronegativity difference. The following subsections discuss these parameters in the given order.

As we already reported in Part I, the *R* elements of the *R*_2_ *T*Si_3_ compounds are either referred to as ionic with oxidation state +II (alkaline earth metals, Eu and Yb) (von Schnering *et al.*, 1996 ▸; Cardoso Gil *et al.*, 1999 ▸) or as metallic (Sc, Y, lanthanides and actinides) (Evers *et al.*, 1977*a* ▸, 1978 ▸, 1980 ▸; Cardoso Gil *et al.*, 1999 ▸; Brutti *et al.*, 2006 ▸). We will adopt this grouping and discuss it accordingly.

### 3.1. Crystal structure   

To characterize the *R*Si_2_ and *R*_2_ *T*Si_3_ compounds, on the one hand we will distinguish them concerning their structure types: hexagonal AlB_2_-like, tetragonal ThSi_2_, orthorhombic AlB_2_-like, and orthorhombic GdSi_2_ and on the other hand by their ordering (ordered or disordered).

As already discussed in Part I, the different lattice types arise for different element combinations. The orthorhombic variants of the AlB_2_-type are only present for divalent *R* elements combined with monovalent *T*. The orthorhombic GdSi_2_-type only arises for lanthanide *R*Si_2_ compounds as intermediate structure between tetragonal ThSi_2_ for *R* elements with lower and hexagonal AlB_2_ for higher atomic number. The ThSi_2_-type also forms for actinide compounds, even with ternary composition, and for Nd_2_AgSi_3_ and Er_2_CuSi_3_. Otherwise, the dominant, hexagonal AlB_2_-type is realized, which indicates that this is the most flexible type.

As we reported in Part I, the compounds of interest exhibit a wide range of ordered structure types. All ordered variants have an AlB_2_-like lattice and exhibit a highly similar structural pattern of [Si_6_] rings isolated by *T* elements, see Fig. 1 ▸(*c*). To characterize these types minimally, we introduce the parameter range of ordering *n*. We define *n* as the number of Si/*T* layers along *c* in the unit cell, illustrated by different colors. If the Si/*T* atoms do not order then *n* equals 0 and we mark this with black in Fig. 2 ▸(*g*). Table 1 ▸ shows the correspondence between the degree of ordering and the different structure types that were introduced in Part I. All AlB_2_-like ortho­rhombic variants possess ordered Si/*T* atoms, hence *n* ≥ 1 applies. Despite the challenging detection and interpretation of satellite reflections, 42.9% of all articles about AlB_2_-like *R*_2_ *T*Si_3_ compounds (79 of 184) report ordered Si/*T* sites, showing the clear tendency of the AlB_2_-type to form ordered structures. The respective box plot in Fig. 3 ▸ shows that the Si/*T* atoms only order for AlB_2_-like compounds.

#### 3.1.1. Systematic lack of Si/*T* ordering   

Most compounds crystallize in both disordered and ordered structure types. However, some *R* and *T* elements seem to hamper the Si/*T* ordering. *R* elements that so far are not known to form any compounds with ordered Si/*T* atoms are Sc, Sr, Pm, Sm, Lu, and Th. However, for further analysis only the Th group is significant, as the others have too few data points. The Th series comprises only three hexagonal compounds (*T* = Co, Ni, Cu), which would have the potential to form ordered structures. As the latest articles concerning Th_2_ *T*Si_3_ were published in 1994 (Albering *et al.*, 1994 ▸) and are thus relatively old, further research concerning possible Si/*T* ordering would be reasonable.

So far, the *T* elements without reported Si/*T* ordering are Al and Ni, with Al having too few data sets for a reliable interpretation. In Section 4.4, the Ni compounds are discussed in more detail.

Despite the lack of possibility to interact with a *T* element, even the disilicides are able to form ordered structures, according to Ji *et al.* (2004 ▸) and Tsai *et al.* (2005 ▸), by interacting with vacancies. The corresponding articles report on non-stoichiometric compounds with formula *R*Si_2−*x*_, thus the Si sublattice contains vacancies, which induce ordering. Section 3.1.4 comprises a discussion concerning the probable electronic reasons for these non-stoichiometric disilicides.

#### 3.1.2. Special case *R* = U   

The research on U_2_ *T*Si_3_ compounds started in the 1990s. In particular, Chevalier *et al.* (1996 ▸), Pöttgen & Kaczorowski (1993 ▸) and Kaczorowski & Noël (1993 ▸) conducted many experiments concerning the structure determination as well as the magnetic and complex susceptibility. About 60% of all U_2_ *T*Si_3_ reports originate from these three authors and only 16% of all reports are from the year 2000 or later.

The overall average of hexagonal compounds with Si/*T* ordering is 42.9%, however, only 23.8% of hexagonal U_2_ *T*Si_3_ compounds have been reported to order. This could originate from the limited hardware and software capabilities at the time of those investigations, which were probably not sensitive enough to detect weak satellite reflections.

Related literature often discusses that the Si/*T* disorder induces randomly frustrated U–U exchange interactions, mediated by a hybridization between electrons of the uranium *f*- and the *T* element’s *d*-orbitals. This hybridization stabilizes a magnetic system with a disordered spin structure (Li *et al.*, 1997 ▸, 1998*b* ▸,*a* ▸, 1999 ▸, 2002*b* ▸, 2003*b* ▸; Kaczorowski & Noël, 1993 ▸; Kimura *et al.*, 1999 ▸). The *f*(U)–*d*(*T*) hybridization only occurs for specific configurations of a U atom with an appropriate *T* element.

For instance, the compound U_2_FeSi_3_ does not seem to provide this configuration, as it was reported with ordered Si/*T* atoms (Yamamura *et al.*, 2006 ▸). So far, U_2_FeSi_3_ is the only *T* = Fe compound with experimental evidence for Si/*T* ordering. For further information on the influence of the electronic structure, please see Section 3.6.3.

#### 3.1.3. Special case *T* = Pd   

Several working groups synthesized and analyzed *R*_2_PdSi_3_ compounds, *e.g.* Szytuła *et al.* (1999); Kotsanidis *et al.* (1990) ▸; Frontzek *et al.* (2006 ▸, 2009 ▸); Behr *et al.* (2008 ▸); Leisegang (2010 ▸); Tang *et al.* (2011 ▸). The only compound among them without ordered Si/*T* atoms is Lu_2_PdSi_3_, which is a rather recent compound with first and only reports from 2013 (Cao *et al.*, 2013*a* ▸,*b* ▸). A more detailed view reveals that the stoichiometry of the analyzed compound is in fact 2.34 : 1 : 3.51 and that therefore the required ratio of *T* and Si is not given.

#### 3.1.4. Si deficiency for *R*Si_2_ compounds   

Since 1959, various authors reported on Si deficiency in the lanthanide disilicides (Brown & Norreys, 1959 ▸, 1961 ▸; Mayer *et al.*, 1962 ▸, 1967 ▸; Houssay *et al.*, 1989 ▸; Auffret *et al.*, 1991 ▸; Kaczorowski & Noël, 1993 ▸; Ji *et al.*, 2004 ▸; Gorbachuk, 2013 ▸; Weitzer *et al.*, 1991 ▸; Baptist *et al.*, 1990 ▸; Dhar *et al.*, 1987 ▸; Eremenko *et al.*, 1995 ▸; Gladyshevskii & Émes-Misenko, 1963 ▸; Iandelli *et al.*, 1979 ▸; Knapp & Picraux, 1986 ▸; Koleshko *et al.*, 1986 ▸; Kotroczo & McColm, 1994 ▸; Land *et al.*, 1965 ▸; Leisegang *et al.*, 2005 ▸; Mulder *et al.*, 1994 ▸; Murashita *et al.*, 1991 ▸; Pierre *et al.*, 1990 ▸, 1988 ▸; Sato *et al.*, 1984 ▸; Weigel & Marquart, 1983 ▸; Weigel *et al.*, 1977 ▸; Yashima *et al.*, 1982*a* ▸,*b* ▸, 1982*c* ▸; Yashima & Satoh, 1982 ▸). For hexagonal compounds, the actual composition is *R*Si_2−*x*_ with *x* ∈ [0.3, 0.4], which corresponds to one missing Si per hexagon *R*_3_Si_5_ (Ji *et al.*, 2004 ▸; Tsai *et al.*, 2005 ▸). For tetragonal compounds, the most frequent composition is *R*Si_1.8_ with 42.1% occurrence, followed by *R*Si_1.9_ with 10.5%, *R*Si_1.73_ with 5.3%, *R*Si_1.85_ with 5.3%, and other undetermined compositions. A composition of *R*Si_1.75_ would accord with one vacant Si per tetragonal unit cell, which would allow ordered, non-stoichiometric, tetragonal structures, see also §4.5.1.

### 3.2. Lattice parameters and Si—*T* distance   

The lattice parameters of the different structure types within *R*Si_2_ and *R*_2_ *T*Si_3_ compounds are not necessarily comparable to each other, due to the different underlying lattices, *e.g.* hexagonal/tetragonal and ordered/disordered. The different structure types of the AlB_2_-like compounds can be interpreted as supercells of the original AlB_2_-type, which thus serves as basis of comparison. Therefore, we define normalized lattice parameters by dividing the lattice parameters of the AlB_2_-like compounds by the multiplicity in the respective direction. Thus, all lattice parameters become comparable with the parameters of the AlB_2_-type. For instance, the Ce_2_CoSi_3_ type consists of two AlB_2_-like cells along the *a* and one along the *c* direction, thus the lattice parameter *a* needs to be divided by 2. Figs. 2 ▸(*a*) and 2 ▸(*b*) show the trend of these normalized lattice parameters.

The box plot of the *a* parameter (see Fig. 3 ▸) shows that the AlB_2_-like *R*Si_2_ compounds have lower *a* values than their ternary counterparts, as their lattice is extended by the larger *T* elements. The lattice parameter *a* of the ThSi_2_ and GdSi_2_ lattices is determined by similar symmetrical components as in AlB_2_-like lattices, *i.e.* the distance between two Si/*T* atoms that are trigonally coordinated to the same central atom. However, by comparing binary or ternary compounds with each other, *a* is mostly larger for ThSi_2_- and GdSi_2_-type structures than for AlB_2_-like structures, as their trigonal coordinations are slightly distorted (Nentwich *et al.*, 2020 ▸), see Fig. 2 ▸(*a*). In general, the *a* parameter is larger for the ThSi_2_-like compounds, as the hexagonal 2D network is less rigid in comparison with the tetragonal 3D network. Generally, the distribution range for binary ThSi_2_-type compounds is larger than for the ones of ternary ThSi_2_-type and binary GdSi_2_-type as the latter groups have less representatives. The median of *a* for all lattice types is almost identical with 4.1 Å, except for the AlB_2_-like disilicides with a median of 3.8 Å. Values lower than 3.9 Å always correspond to hexagonal disilicides.

The *b* parameter does not have to be considered separately, as the lattice parameters *a* and *b* are always identical in (quasi) hexagonal compounds and as the difference between both directions is negligible (2.2% in average, *b* always being the bigger one) for (quasi) tetragonal composites.

The *c* parameter of compounds with ThSi_2_ and GdSi_2_ structure types is not related in any way to the *c* parameter of the ones with AlB_2_-like structure type as the underlying symmetry is completely different, see Fig. 1 ▸. The radius of the *R* element is the *c*-determining factor of the AlB_2_-like compounds as the Si sublayers are connected by weak van der Waals forces. Here, the average value is approximately 4.1 Å with slightly higher values for orthorhombic AlB_2_-like compounds, see Fig. 2 ▸(*b*) and Fig. 3 ▸. For ThSi_2_-like compounds, *c* is determined by the Si—*T* distance within the trigonal coordination [*c* ≈ 2(3)^1/2^ *a*], see equation (1). The mean values of *c* for ThSi_2_- and GdSi_2_-like compounds are 13.8 Å and 13.4 Å, respectively. The *c* parameters of the compounds with orthorhombic GdSi_2_ lattice distribute over a very narrow range of 13.22–13.94 Å, only exceeded by the outlier LaSi_2_ (Mayer *et al.*, 1967 ▸), with La being the biggest *R* element within the GdSi_2_ compounds. The presence or absence of a *T* element causes more pronounced effects of the *c* parameter for ThSi_2_-like compounds than for AlB_2_-like compounds. In contrast, the *a* parameter is more sensitive for AlB_2_-like compounds.

Because of these predominantly symmetry-related variations of the lattice parameters, we decided to employ further types of measures: a modified *c*/*a* ratio and the smallest Si—*T* distance *d*.

The *c*/*a* ratio is often used in relation with AlB_2_-type structures. To enable comparability between the structure types with different ranges of ordering, we redefined *c*/*a* as the ratio of minimal *R*–*R* distances along the *c* direction and within the *a*,*b* plane: *d*(*R*,*R*)_*c*/*d*_(*R*,*R*)_*a*,*b*_ for compounds with AlB_2_-like lattice. This redefined *c*/*a* ratio characterizes the changes of the prototypic AlB_2_-like cell for all *R*Si_2_ and *R*_2_ *T*Si_3_ compounds, as originally intended.

Fig. 2 ▸(*c*) shows the resulting *R*–*T* diagram for the *c*/*a* ratio. The related box plot in Fig. 3 ▸ indicates that the ternary AlB_2_-like compounds behave similarly to each other, with an average of 1.04 and values between 0.95 and 1.14. The binary AlB_2_-like compounds have a similar range, but with a strong tendency for a ratio of 1.08 as the narrow percentiles indicate. The *c*/*a* ratios of compounds with ThSi_2_ lattices have a very large spread (3.08–3.61), which emphasizes the aforementioned flexibility of the Si sublattice in those compounds. In contrast, the *c*/*a* ratios of GdSi_2_ lattices correspond approximately to the average value of 3.30. This smaller range is caused by the very low amount of compounds with GdSi_2_ lattice, *i.e.* only lanthanide disilicides, thus the incorporated *R* elements have very similar chemical and sterical properties.

We determined the second type of measure, the shortest Si—*T* distance *d*, from the (normalized) lattice parameters *a* and *c* by applying a formula by Mayer *et al.* (1962 ▸) that was originally only used for disilicides and utilizes the symmetries of the underlying Si/*T* sites: 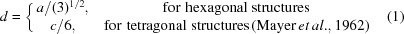
For compounds with a ThSi_2_ lattice, this formula assumes that the bonds along the *c* direction (interchain) are shorter than the bonds roughly along *a* and *b* direction (intrachain). For the compounds with AlB_2_-like symmetry and buckled Si/*T* sublattice, the values for *d* are underestimated by up to 5.6%. Nevertheless, we applied this formula to all tetragonal datasets as many reports only give lattice parameters but no Wyckoff positions. Thus, an exact determination of *d* is not possible. For similar reasons, we also applied this formula to compounds with orthorhombic AlB_2_-like and orthorhombic GdSi_2_ lattices. Fig. 2 ▸(*d*) shows the results in an *R*–*T* plot. As already discussed for the lattice parameters, the distances within the compounds decrease with increasing atomic number of *R* and with decreasing period of *T*. Both is indirectly related with the radii of the contained elements.

The box plot in Fig. 3 ▸ shows that the distance *d* is lower for binary compounds than for ternary ones, which indicates the lattice spread by the *T* elements.

#### 3.2.1. Structure determined by *d*?   

Mayer *et al.* (1962 ▸) found a relation between the shortest Si–Si distances and the symmetry of lanthanide disilicides. They stated that a specific crystal system arises in a unique range of *d*-values, as listed in Table 2 ▸. However, this grouping is not applicable to the *R*Si_2_ compounds in general, as our data base shows wider ranges of *d* for the different lattice types. Additionally, the box plot in Fig. 3 ▸ shows clearly that the *d* values of *R*Si_2_ and *R*_2_ *T*Si_3_ compounds exceeds the limits given by Mayer *et al.* (1962 ▸). Hence, the limits found by Mayer *et al.* (1962 ▸) were a consequence of the choice of the examined disilicides and are not applicable to the *R*Si_2_ and *R*_2_ *T*Si_3_ compounds in general.

We also learn from the box plots that compounds with orthorhombic AlB_2_-like symmetry have the largest Si—*T* distances *d*, as the incorporated *R* elements have the biggest radii. Compounds with GdSi_2_ symmetry have the lowest *d*. Values below 2.0 Å only appear for hexagonal systems.

### 3.3. Thermal treatment   

Chevalier *et al.* (1983 ▸) discovered the Si/*T* ordering within the *R*Si_2_ and *R*_2_ *T*Si_3_ compounds after applying a thermal treatment to those compounds, for the first time. The occurrence of ordering might be strongly dependent on the thermodynamics of the growth process. For instance, if the kinetic barrier for atomic rearrangement is reached during cooling, then the ordered structure might not be sufficiently stabilized and might not form. Therefore, a subsequent thermal treatment of the crystals might be essential to reach the thermodynamic ground state. The smaller the differences in the formation energy between the ordered and disordered structural variants, the weaker are the driving forces within the ordering process and the longer the necessary thermal treatment.

The most common approach reported in literature is the constant heating of the whole sample for a certain time. Additionally, we categorize the floating zone method (Behr *et al.*, 2008 ▸) as a second type of thermal treatment, as the effect on the atomic ordering is comparable. Fig. 2 ▸(*h*) visualizes the treatment with respect to the applied method (floating zone – filled circle, constant heating – open circle, none – ×), the corresponding temperature (color), and duration (circle size). Only for very few compounds we did not find experimental reports with thermally treated samples. Among them are *R*_2_NiSi_3_ compounds, and disilicides with *R* being an alkaline earth metal or an actinide. The correlation of the thermal treatment and Si/*T* ordering is discussed in §4.4.

### 3.4. Element radii and their ratio   

**Figure 13:**
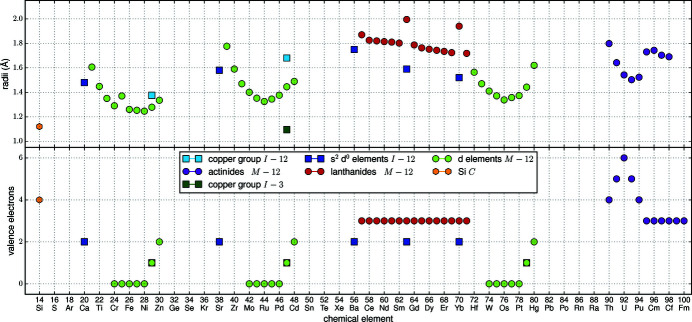
Valence electrons and radii for the chemical elements up to atomic number 100. Determination of the valence electrons based on electronic configurations from Holleman & Wiberg (2007 ▸) and considerations from §3.6. Atomic radii also from Holleman & Wiberg (2007 ▸). The groups of elements that are relevant during this work are highlighted with different colors and shapes.

Following Hume-Rothery & Raynor (1962 ▸), atoms can replace each other if their radii differ by only ±15%. To consider this limit, the correct determination of the radii is essential. The terms of isotropy, coordination, and charge number characterize the type of radius, and thus the adequate size. For simplicity, we consider all atoms and ions to be isotropic (hard sphere approach), and further influences to be electronic in nature. This approach allows screening a great variety of compounds with little computational effort, but is rather inaccurate for Si atoms, therefore we performed complementary Bader analyzes for a selection of representative structures, see §3.6.2. The other two terms need to be considered separately for every element. Fig. 13 ▸ summarizes the radii chosen within this work and Appendix A[App appa] explains our choices.

Mayer *et al.* (1967 ▸) studied the dimorphism of selected lanthanide disilicides by evaluating the ratio of radii *q*_rad_ = *r*_Si_/*r*_*R*_. In order to apply this formula to the *R*_2_ *T*Si_3_ compounds, the calculation has to be extended for the *T* element. We used a weighted average for the Si/*T* position and received: 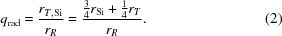
That purely theoretical ratio of radii *q*_rad_ was calculated for all points of the diagram Fig. 2 ▸(*e*). By analyzing the color distribution, none of the hypothetical compounds appears to be instable as the *q*_rad_ values of the already reported compounds comprise the values of all hypothetical compounds. The box plot of *q*_rad_ in Fig. 3 ▸ reveals that the average value of all lattice types is 0.64. Additionally, the quartiles are also very similar for the AlB_2_-like and the binary ThSi_2_-type compounds with 0.61 and 0.65, which seems to be the most stable ratio. In §4.3.2 correlations of *q*_rad_ and the structure type are discussed.

#### 3.4.1. Laves phases   

The ratio of radii *q*_rad_ allows the evaluation of the *R*Si_2_ and *R*_2_ *T*Si_3_ compounds with respect to the restrictions that have to be met by Laves phases. These phases have the sum formula 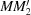, with two metals *M* and *M*′, whose radii yield *r*_*M*_ : *r*_*M*′_ = *r*_*R*_ : *r*_*T*, Si_ ≈ 1.225. Given a 10% tolerance, the formula is only valid for compounds with *R* = U, Np, Pu, which, however, do not crystallize in the structure types that are typical for the Laves phases, *e.g.* MgCu_2_ [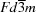 (*b*,*c*)], MgZn_2_ [*P*6_3_/*mmc* (*a*,*f*,*h*)], or MgNi_2_ [*P*6_3_/*mmc* (*e*,*f*,*f*,*g*,*h*)]. Therefore, we conclude that the *R*Si_2_ and *R*_2_ *T*Si_3_ compounds do not belong to the Laves phases.

### 3.5. Density and atomic packing factor   

The density was calculated based on the reported lattice parameters and the listed atomic masses of the included elements. The density of tetragonal and hexagonal variants of the same composition are almost identical, see Table 3 ▸. Thus, *R* and *T* elements occupy approximately the same volume in the different lattices. In Section 4.5.2, a closer analysis concerning the occupied volume of the *R* elements is performed. For *R*Si_2_ compounds, the density of the hexagonal arrangement is slightly higher than the tetragonal one (on average ≈ 1.42%), especially for the actinide compounds (−3.70% and −4.76% for U and Th, respectively). In contrast, for the ternary *R*_2_ *T*Si_3_ compounds, the density of the tetragonal arrangement is slightly higher than for the hexagonal one.

The atomic packing factor apf is defined as the ratio of the whole particle volume to the volume of the unit cell 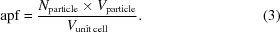
The apf is maximal if big atoms form a frame and smaller atoms fit perfectly into the gaps in between. The volume of the atoms is determined by their radius, thus the apf is strongly dependent on the choice of the radius. As we mentioned before in §3.4, the accurate determination of the correct radius is challenging. Figs. 2 ▸(*h*) and 3 ▸ show the apf when we assume the metallic twelvefold coordinated radii for all elements. The average apf is 0.65, but with large deviations from 0.45 to 0.85 (NpSi_2_ and YbSi_2_, respectively) for all symmetries but orthorhombic GdSi_2_. These small variations for GdSi_2_-like compounds originate from their highly similar chemical composition, namely binary disilicides with lanthanides of the intermediate range. These *R* elements have very similar radii and very similar chemical properties, thus, also the apf is expected to be very similar. The lowest apf arises for actinide compounds, as the huge *R* atoms determine the lattice parameters and the Si atoms are too small to fill the resulting spaces. The highest apf arises for compounds with divalent *R* elements.

In contrast, if we applied the ionic radii to the divalent *R* elements, the respective compounds would exhibit an average apf and the disilicide compounds would have the highest apf. This supports the expectation that the binary silicides should have the largest apf as the incorporation of a *T* element enlarges the particle volume (from Si to transition metal), but to a greater extent also the unit-cell volume.

### 3.6. Electronic structure   

The electronic structure is a crucial factor for local atomic ordering and for the suitability of an element to replace another one in a given structure. The characterization of the electronic structure is challenging, thus to gain a thorough understanding, we combined different approaches of varying complexity, in particular geometric bond network, principle of hard and soft acids and bases (HSAB), valence electron concentration (vec) analysis, Bader analysis, and molecular orbital (MO) theory.

Following Hume-Rothery & Raynor (1962 ▸), the valence electron concentration is defined as ratio of the number of valence electrons to the number of atoms. The vec is mostly used in context with the Hume-Rothery phases, but has already been discussed for some *R*Si_2_ and *R*_2_ *T*Si_3_ compounds (Cardoso Gil *et al.*, 1999 ▸; Chevalier *et al.*, 1984 ▸, 1986 ▸; Gorbachuk, 2013 ▸; Mayer & Felner, 1973*b* ▸,*a* ▸; Rieger & Parthé, 1969 ▸; von Schnering *et al.*, 1996 ▸). Partially, these discussions only evaluated the vec of the Si/*T* sublattice (Cardoso Gil *et al.*, 1999 ▸; von Schnering *et al.*, 1996 ▸), thereby neglecting the electronic influence of the *R* element. However, we will show in §4.5.2 that the electronic influence of the *R* element is evident when discussing the complete range of existing *R*Si_2_ and *R*_2_ *T*Si_3_ compounds.

We evaluated the vec for the *R*Si_2_ and *R*_2_ *T*Si_3_ compounds as stated in Appendix B.1[App appb]

#### 3.6.1. Geometric bond network   

At first, we will analyze the electronic structure from a geometrical point of view. In both ThSi_2_- and AlB_2_-like structures, each Si/*T* atom is surrounded by three other Si/*T* atoms in a planar trigonal coordination. This corresponds to an *sp* ^2^ hybridization and a conjugated π electron system. Ideally, all Si—*T* bond lengths should be equidistant, the bond angle should be 120°. Every Si atom possesses a *p*_*z*_ orbital perpendicular to the trigonal plane. In the hexagonal arrangement, the Si/*T* sublattice forms graphene-like layers. Thus, all *p*_*z*_ have the same orientation and form a π electron system in 2D. In the tetragonal arrangement, the Si/*T* atoms form zigzag chains (intrachain bonds). These chains point roughly along *a* and *b* direction, alternately, and they are connected by interchain bonds along *c*. Hence, only *p*_*z*_ orbitals of Si atoms within the same chain face each other and can build a π system. Thus, the π electron system only assembles in 1D, but alternating between *a* and *b* direction, along the *c* stacking. The combination of an ‘ideal lattice’ (Nentwich *et al.*, 2020 ▸) with a reasonable distribution of double bonds to this lattice results in shorter π intrachain bonds in tetragonal systems, in contrast to equidistant lengths for all directions in hexagonal systems.

Next, we will examine the structural boundary conditions on the ability of the Si sublattice to buffer electrons, independently from the choice of the *R* or *T* element. This ability mainly depends on the presence or absence of a *T* element. To discuss the delocalized double bonds, the smallest geometrical unit of interest is the [Si_6_] ring.

Depending on the state of the *R* element, we can now determine the valence electron number of the *T* element related to a certain valence electron amount (vea) within the [Si_6_] ring 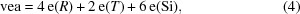
with the charge transfer number e(*x*) of the metal elements *x* according to their formal oxidation states. In this first estimation of the charge distribution, we restrict our assumptions to integer oxidation states for the *R* element, although this is not mandatory. Table 4 ▸ gives an overview of the possible electronic contributions of the *T* elements, considering a given valence electron amount in the [Si_6_] ring and a certain state of the *R* element.

**Figure 14:**
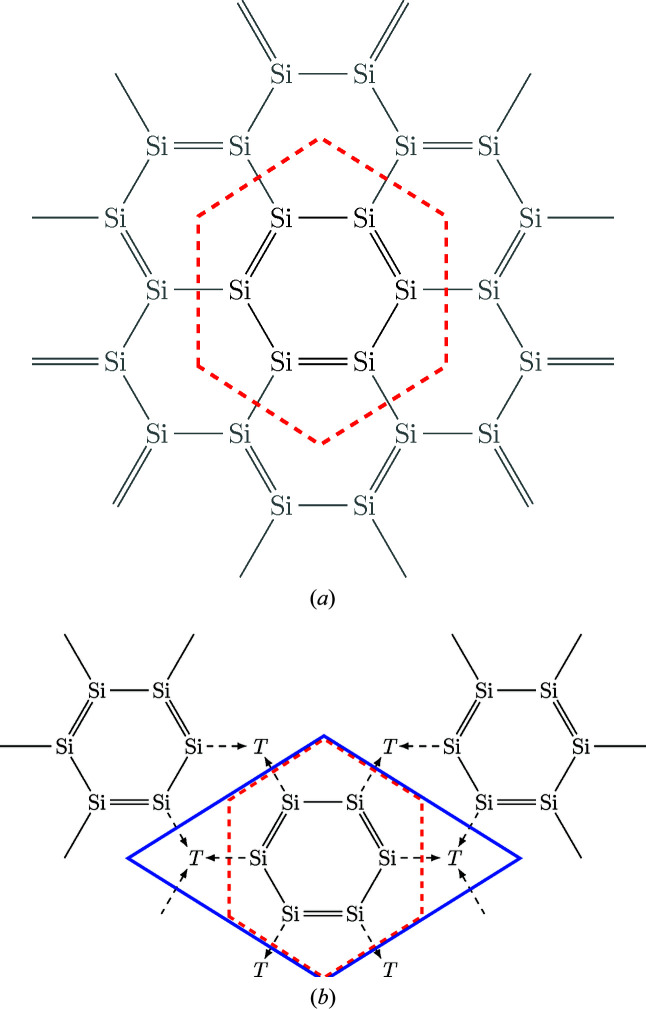
Snapshot of the distribution of delocalized single and double bonds in AlB_2_-like *R*Si_2_ and ordered *R*_2_ *T*Si_3_ compounds. (*a*) *R*Si_2_ compounds and (*b*) ordered *R*_2_ *T*Si_3_ compounds. These figures only include the Si/*T* sublayers. The unit cell of the minimal structure pattern of ordered *R*_2_ *T*Si_3_ compounds is highlighted in blue and the isolated Si hexagon is highlighted in red. Dashed arrows indicate the coordinative bonds between Si and *T* atoms.

For metallic hexagonal *R*Si_2_ compounds, the [Si_6_] ring contains nominally 24 electrons (three times six from σ bonds and three times two from π bonds) corresponding to four electrons per Si, meaning a neutral state, see Fig. 14 ▸(*a*). Figs. 14 ▸(*a*) and 14 ▸(*b*) represent a snapshot of the distribution of the single and delocalized double bonds to the lattice, with symmetrically equivalent Si positions. As these figures are snapshots, the distribution of the single and double bonds will be different at another moment of time. The feasibility of a consistent distribution is important here, as well as the charges of the Si atoms. In general, we also expect a neutral state of Si for the binary tetragonal compounds, because of the similar, local, planar threefold symmetry as for the hexagonal *R*Si_2_ compounds. However, one article about ionic tetragonal disilicides exists concerning EuSi_2_ (Evers *et al.*, 1977*a* ▸), hence each Si atom should have a single negative charge.

For ordered, metallic *R*_2_ *T*Si_3_ compounds the *R* element accounts for partial charge transfer [e(*R*) < 2] to the Si ring with remaining valence electrons, which potentially contribute to the delocalized electron gas of the metal, see introduction of §3. In general, the Si ring contains nominally 30 electrons (two times six from coordinative bonds, two times six from σ bonds, and six from π bonds), which means that the Si atoms form polyanionic [Si_6_]^−6^ rings. The elements in polyanions often behave like elements of the next higher group of the periodic table. Here, the structure of the Si sublattice resembles the structure of black phosphorus, see §3.6.4. The remaining electrons needed for a stable configuration must be provided by the *T* element. For configurations with high electron amounts a very high oxidation state of the *T* element follows. Only elements that can provide that sufficient amount of electrons are expected to be incorporated in the respective compound. The excess electrons of *T* elements that provide more electrons than needed will contribute to the electron gas, *e.g.* in U_2_MnSi_3_. The electronic stabilization of the Si rings and respective charge transfer will be balanced by both the *R* and the *T* element, depending on the ionization energies of the *R* and the *T* species. Table 4 ▸ shows that this reasoning would exclude neutral *R* elements. Realistic metallic configurations are thus formally represented by slightly charged *R* elements (*R* ^+*x*^, 0 < *x* < 2). These compounds possess a covalent bond network, and no ionic character.

The +II oxidation state [e(*R*) = 2] of the alkaline earth metals as well as of Eu and Yb is a special case and represents an ionic state with full charge transfer of the outer valence shells. For this group, a [Si_6_]^10−^ ring was reported (von Schnering *et al.*, 1996 ▸; Cardoso Gil *et al.*, 1999 ▸; Peter *et al.*, 2013 ▸; Zeiringer *et al.*, 2015 ▸), which corresponds to 34 electrons and a nominal charge of −1.67 for Si, see §3.6.5. We already discovered that this configuration only arises for the divalent *R* elements in combination with the noble metals Ag and Au. The elements Ag and Au prefer the +I oxidation state, in contrast to +II from *e.g.* Cu. Additionally, the divalent state of the *R* elements has only been reported for ionic compounds and only in combination with monovalent *T* element. Hence, other vea configurations with a divalent *R* can be excluded.

Because of its intermediate position between the 30 and 34 electron configuration, the 32 electron ring is also expected to be stable (even though we will show that it is less stable than the aforementioned ones, see §3.6.5). For lower vea values, the *T* elements need to contribute less. Even a 26 electron configuration with no contributions by the *R* element and 1 electron from each *T* element would be possible. However, lower electronic configurations can be excluded for *R*Si_2_ and *R*_2_ *T*Si_3_ compounds. Analogously, the *T* elements need to contribute more for higher electron configurations. For the metallic 36 electron configuration, the *T* element needs to account for four or six electrons, which would already result in ionized *T* elements and thus ionic compounds. Hence, realistic electron configurations possess between 28 and 34 electrons.

**Figure 15:**
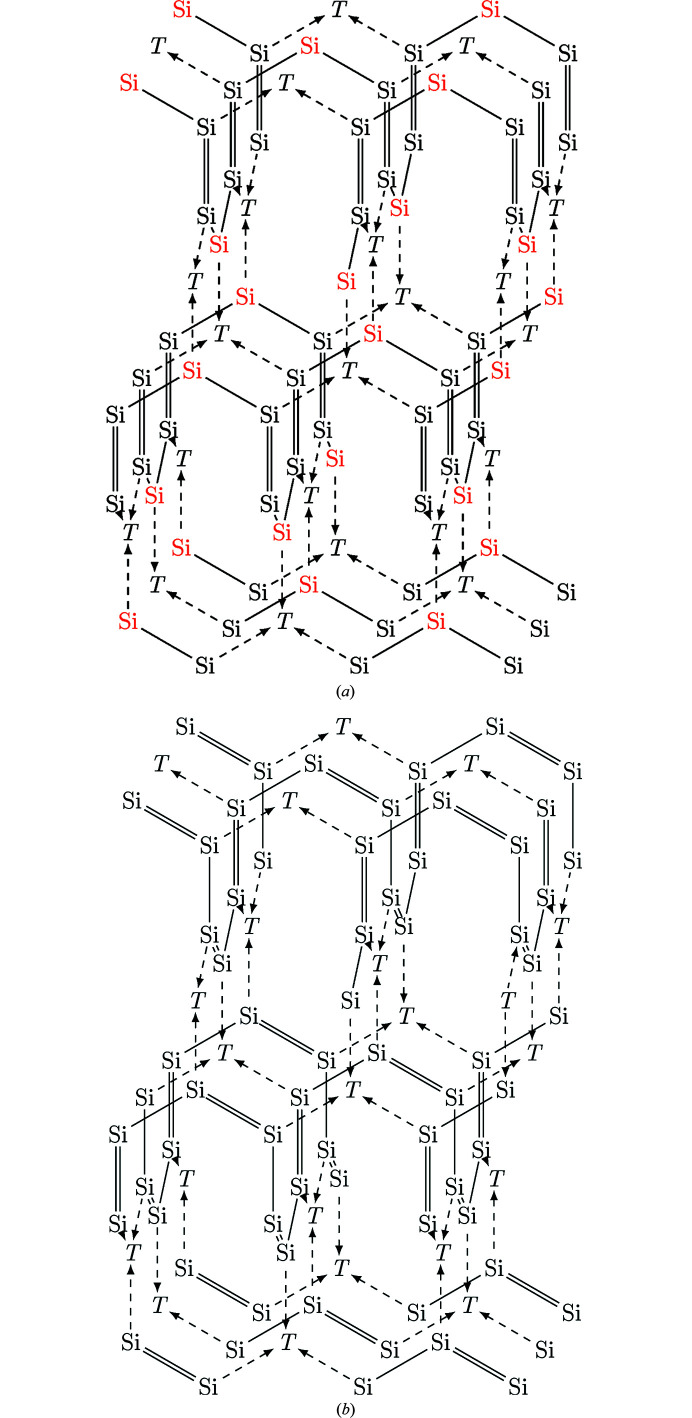
Distribution of single and double bonds in *R*_2_ *T*Si_3_ compounds with the proposed tetragonal structure with Si/*T* ordering: (*a*) double bonds only in interchain direction and (*b*) double bonds in inter- and intrachain direction These figures only include the Si/*T* sublattice. Dashed arrows indicate the coordinative bonds between Si and *T* atoms. The Si highlighted in red are neutral, the black Si have a charge of −I.

**Potential ordering in tetragonal structures**. Part I of this work described the construction of a tetragonal ThSi_2_-like structure with Si/*T* ordering from a geometrical point of view (Nentwich *et al.*, 2020 ▸). Here, we try to distribute Si=Si double bonds within this geometric network. We started with the placement of the coordinative bonds between Si and *T* as in the AlB_2_-like compounds. Now, we can place arbitrarily a double bond on an interchain bond. The next double bond can either be placed onto another interchain bond or onto an intrachain bond. Depending on this choice, two different models arise, shown in Figs. 15 ▸(*a*) and 15 ▸(*b*), respectively. The model in Fig. 15 ▸(*a*) allows an arrangement with different electronic configurations of Si atoms on different Wyckoff positions. When Si atoms are connected to each other along the *c* direction, they share a double bond. Additionally, the Si of this Wyckoff site possess a single bond to a second Si and a coordinative bond to a *T* atom. Thus, these Si atoms have eight bonding electrons (four from the double bonds, two each from single and coordinative bond) and are counted as Si^−I^ as both electrons from the coordinative bond contribute to the formal charge of Si. The Si atoms which are connected to a *T* element along the *c* direction, possess this coordinative bond and two single bonds to other Si atoms. Thus, these Si atoms have six bonding electrons and are neutral. The Si atoms of the second model in Fig. 15 ▸(*b*) are all electronically equivalent and are negatively charged.

#### 3.6.2. Bader analysis   

By performing a Bader analysis for selected *R*Si_2_ and *R*_2_ *T*Si_3_ compounds of different structure types, we tried to reveal the influence of the electronic structure onto the lattice and the Si/*T* ordering. The calculations comprise Nd_2_ *T*Si_3_ compounds, including the proposed ordered tetragonal structure (POTS) Nd_2_AgSi_3_. We also modeled a hexagonal version of Nd_2_AgSi_3_ to compare the influence of the lattice onto the charge of the individual atoms. Additionally, we calculated Nd_2_PdSi_3_ as a representative of compounds with a non-monovalent *T* element, as we assume special electronic conditions involved with noble metals such as Ag. Further, we chose Nd_2_CuSi_3_ and Nd_2_NiSi_3_ to evaluate the influence of the *T* element’s period (Ni → Pd and Cu → Ag). And finally, we evaluated the two structure types AlB_2_ and ThSi_2_ for the disilicide NdSi_2_.

Table 5 ▸ gives an overview of the calculated Bader charges and the Bader volumes as well as the tabulated electronegativity values according to the Pauling scale (Lide, 2010 ▸). All the calculations are given for the same supercell size, which means for the same amount of atoms, *R*_2_ *T*Si_3_ or *R*_2_Si_4_, respectively. The Bader charges of different atoms of the same element within the same compound may differ from each other depending on the corresponding Bader volume. The larger the volume of an atom, the more electron density is attributed to this atom, see Appendix C[App appc]. Therefore, the evaluation of the average volumes and charges is sufficient.

In contrast to the theoretical considerations of the previous paragraph, the DFT-based Bader analysis considers partial electron transfer in a picture of electron density distributions.

The calculations for both structure types of the disilicide NdSi_2_ yield the same charges for Si and Nd of ≈ −0.6 and ≈ 1.2, respectively. The formation energies of both structure types indicate that with −4.20 eV the hexagonal lattice is more stable than the tetragonal one with −3.97 eV (Nentwich *et al.*, 2020 ▸). However, this is not in accordance with the fact, that we did not find reports about hexagonal NdSi_2_. Furthermore, the calculated charges for both structures indicate an ionic charge transfer, which accords with considerations about Zintl phases, given later in §3.6.4.

The calculation for hexagonal Nd_2_AgSi_3_ reveals a Si charge of ≈ −0.58, which is rather low compared to the formal charges for isolated Si hexagons (vea = 26 means a charge of 0.3 and vea = 32 means a charge of 1.3) presented previously. The former discussion was based on the strict assumption that all electrons are localized. In contrast, the Bader analysis considers contributions of the electron gas.

The structural relaxation of POTS Nd_2_AgSi_3_ shows that the Si—*T* and Si—Si distances deviate strongly (up to 6.4%), see Table 6 ▸. As expected, the weak Si—*T* coordinative bonds are elongated compared to the covalent Si—Si bonds. Furthermore, the Si—Si distances are also not equal, the *d*_inter_(Si, Si) bonds along the *c* direction (interchain) are slightly elongated compared to the intrachain direction *d*_intra_(Si, Si). This seems to contradict the original description of the tetragonal Si network as constructed from shorter inter- and longer intrachain bonds. However, the ordered arrangement of the *T* atoms changes the boundary conditions and causes slightly different arrangements to become energetically favored over the disordered variants. These length distributions contradict the model in Fig. 15 ▸(*b*) and strengthen the model in Fig. 15 ▸(*a*).

We also observed that the charges of all elements are comparable in the hexagonal and tetragonal settings of Nd_2_AgSi_3_. This accords with the results for tetragonal and hexagonal NdSi_2_.

For Nd_2_CuSi_3_, we considered the reported structure type (Yubuta *et al.*, 2009 ▸) Er_2_RhSi_3_ (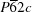, No. 190) and additionally the high-symmetry type Ce_2_CoSi_3_ (*P*6/*mmm*, No. 191). The charges of both structure types are almost identical to each other (≈ 1.26 for *R*, ≈ −0.6 for *T*, ≈ −0.6 for Si). Comparing both Nd_2_CuSi_3_ models with the hexagonal model of Nd_2_AgSi_3_, the charges on *R* as well as on Si are similar (1.26 and 1.29 as well as −0.63 and −0.58, respectively). However, the *T* element is more negatively charged for Nd_2_AgSi_3_, as the radius of Ag is larger than that of Cu and thus the ascribed volume of electron density is larger, see also Appendix C[App appc].

The Si charge of Nd_2_PdSi_3_ in structure type Ce_2_CoSi_3_ (Li *et al.*, 2003*a* ▸; Szytuła *et al.*, 1999 ▸; Xu *et al.*, 2011 ▸) is −0.50, which is the lowest Si charge within the tested range. Compared to all other compounds, the *T* element of Nd_2_PdSi_3_ is the most negative one with −1.14 and the *R* element is one of the most positive ones with 1.32. As the Bader volumes of both *T* elements Pd and Ag are very similar with 23.1 Å and 23.0 Å, respectively, the higher attractiveness of Pd is caused by its higher electronegativity, see Table 5 ▸.

Nd_2_NiSi_3_ exhibits the structure type Ce_2_CoSi_3_ (No. 191) (Felner & Schieber, 1973 ▸; Gladyshevskii & Bodak, 1965 ▸; Mayer & Felner, 1972 ▸, 1973*b* ▸). The charge of Nd is 1.33, which is comparable with Nd_2_PdSi_3_ and also with Nd_2_AgSi_3_ or Nd_2_CuSi_3_. The Si charge is −0.55, which is the second lowest value among the Nd_2_ *T*Si_3_ compounds listed here, but still similar to Nd_2_PdSi_3_. The charge of Ni is −0.76, which is less negative than that of Ag in both Nd_2_AgSi_3_ variants. Again, the reason is the smaller Bader volume of Ni in Nd_2_NiSi_3_ compared to that of Ag in Nd_2_AgSi_3_ at almost identical electronegativity values.

**Figure 16:**
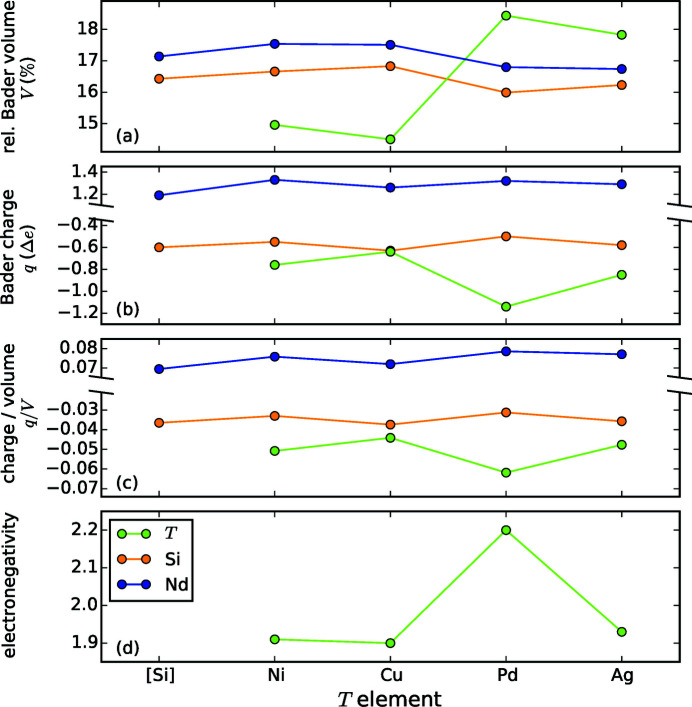
The different charges of Si and the *R* element are caused by the different Bader volumes ascribed to the elements as well as the different electronegativities of *T*.

The atomic radii of all constituents of the AlB_2_-like compounds change depending on the *T* element, as depicted by the Bader volume in Fig. 16 ▸(*a*). The *R* and Si atoms follow the same trend, indicating that the influence of the *T* element affects both equally. This effect is very similar for the Bader charges, see Fig. 16 ▸(*b*). When the charge per volume in Fig. 16 ▸(*c*) is considered, the influence of the *T* element becomes weaker. The largest remaining deviation is for Pd, which also has a very different electronegativity value compared to the other *T* elements, see Fig. 16 ▸(*d*). A high electronegativity means that the corresponding element strongly attracts electrons. Therefore, the electron density within the Bader volume and thus the Bader charge of Pd is larger in comparison with the other *T* elements.

In summary, comparing the results of the Bader analysis for the selected Nd_2_ *T*Si_3_ compounds, the influence of the *T* element’s Bader volume and electronegativity are evident. Within the investigated series, the *R* elements of all models span a narrow range of Bader charges of [1.2, 1.3], confirming our assumptions from the previous paragraph and the general metallic character of the *R*_2_ *T*Si_3_ compounds. Furthermore, we recognized that in all the tested ternary, AlB_2_-like compounds the *R* elements of different Wyckoff sites may exhibit different charges. *R* elements without any *T* element in their first coordination shell exhibit slightly higher charges than those *R* elements with an adjacent *T*. For the latter, *R* is nearly neutral with values between 0.0 electrons for Nd_2_CuSi_3_ and 0.1 electrons for Nd_2_PdSi_3_. A normalization to the respective Bader volume even enhanced the differences in charge. Hence, these differences are solely related to the different Wyckoff site. Deviations of the average Bader charge for Si are comparably small, whereas the span of the *T* element’s charge varies much more, with a deviation of up to half an electron. Thus, in terms of the Bader results the *T* element’s charge is the most sensitive parameter and will be discussed in detail in the following section.

#### 3.6.3. Molecular orbital theory   

Molecular orbital (MO) theory is a versatile tool that, for example, allows the description of the electron localization within a molecule. In general, a complex is a molecular entity consisting of two or more parts that are weakly bonded to each other (weaker than covalent bonds) (Nič *et al.*, 2009 ▸). Although the present *R*Si_2_ and *R*_2_ *T*Si_3_ compounds form infinite networks rather than molecules, the underlying geometry resembles the one of the *ML*_3_, consisting of the central metal *M* and three identical ligands *L*. Thus, we will discuss this approach as alternative bonding variant to the coordinative Si—*T* bonds after introducing the MO of the complex itself.

**Figure 17:**
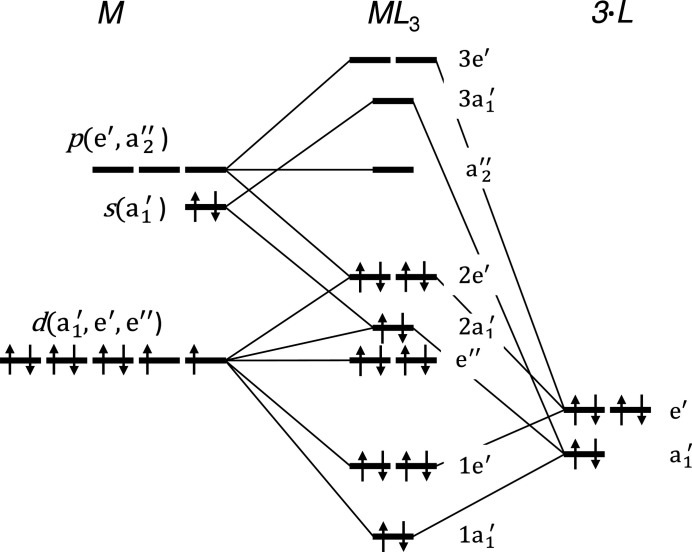
Distribution of the electrons in the *ML*_3_ complex with respect to the *molecular orbital theory* (MO theory) following Jean *et al.* (1993 ▸). Electronic contribution of the metal *M* on the left, contribution of the ligands *L* on the right, molecular orbitals in the middle.

The trigonal-planar *ML*_3_ complex is perfectly stable, if the constituents supply 16 electrons e^−^, with typically 10 e^−^ from the metal *M* (Jean, 2008 ▸), illustrated by black arrows in Fig. 17 ▸. This complex violates the 18-electron rule, which emphasizes the stability of complexes with 18 valence electrons (Holleman & Wiberg, 2007 ▸); however in trigonal planar geometry, the two missing electrons would occupy the non-bonding orbital 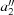, which would not contribute to the stability of this complex (Jean, 2008 ▸).

The central particle is either neutral or positively charged, the ligands are mainly anionic or neutral. The complex itself may be charged as a part of a larger structure, *e.g.* PdH_3_^3−^ (Olofsson-Mårtensson *et al.*, 2000 ▸).

Here, the *T* element is comparable to the metal *M* and the adjacent Si atoms to the ligands *L*. Simplified, three configurations of the [Si_6_] rings can be discussed: entirely formed by double bonds, entirely made of single bonds, or an alternation of both. However, the first configuration would not be stable and can thus be neglected. The three Si atoms could contribute with 6 e^−^ or 3 e^−^ to the complex, respectively, for the remaining two configurations. The electronic contribution of the *T* element depends on its chemical group. For the reported elements these are 7 e^−^ to 11 e^−^ (Mn group to Cu group, respectively). Considering charge transfer between the constituents, even the *R* element will indirectly contribute to the electronic occupation of the complex, in accordance with the example Na^+^Ba^2+^[PdH_3_]^3−^ (Olofsson-Mårtensson *et al.*, 2000 ▸). The remaining valence electrons from the *R* element are assumed to be delocalized forming an electron gas, which is in accordance with experimental observations of metallic conductivity for most of the compounds. This concept of an electron gas coexisting with a complex goes beyond MO theory. However, it does reveal electronic boundary conditions for *T* and *R* elements, which are reasonable with the experimental observations.

So far, we have preferred to discuss the Si network with alternating single and double bonds. In this case, the *R* elements need to contribute with 1 e^−^ to 3 e^−^ each to the complex (Cu and Mn group, respectively). For the case of the Mn group all valence electrons of the *R* element would be consumed. Thus, elements of the Mn group would form the lower limit and compounds with *T* elements of the Cr group or lower cannot be expected. Considering the other side of the elemental range, the elements of the Zn group should also present valid metals for the *ML*_3_ complex. In fact, we found reports about *R*_2_ZnSi_3_ compounds, but only at elevated temperatures. Non-ambient conditions are beyond the scope of this article.

In the case of singly bonded [Si_6_] rings, the elements of the Cu group would contribute with its *d* ^10^ electrons to the complex and with its *s* ^1^ electron to the electron gas. In contrast, the *R* elements would supply 0 e^−^ to 3 e^−^ to the complex for the other groups. For this configuration, elements from the Cr group or lower may be incorporated, but have not been reported yet.

These assumptions suggest that compounds with *T* elements from the Mn group would also be stable. However, we only found two reports on Mn compounds, namely Th_2_MnSi_3_ (Albering *et al.*, 1994 ▸) and U_2_MnSi_3_ (Chevalier *et al.*, 1996 ▸), and none for the *T* elements Tc or Re (in the case of Tc its sparsity could be another cause). In the respective MO state, Mn has to be present in the neutral state. However, in complex compounds, Mn strongly prefers the ionic state, especially +II, +IV, and +VII, over the neutral state (Holleman & Wiberg, 2007 ▸). We assume that U_2_MnSi_3_ is still stable due to a hybridization between *f*(U) and *d*(Mn) electrons, see §3.1.2. Therefore, we expect that the same type of hybridization also occurs in Th compounds such as Th_2_MnSi_3_. Furthermore, this hybridization may also exist in (U, Th)_2_(Tc, Re)Si_3_ compounds. The synthesis of these compounds seems to be promising.

The considerations of the MO theory are completely valid for ordered AlB_2_-like structures. For disordered structures additional low-symmetry arrangements of nearest neighbors arise, due to adjacent *T* atoms in the first neighbor shell. For the ordered tetragonal structure, the Si site splits into two very different environments, which would need to be considered separately.

#### 3.6.4. The formal coordination number and Zintl phases   

The Zintl phases *A*_*x*_ *B*_*y*_ are characterized by a high difference in electronegativity Δ EN(*A*, *B*) = |EN(*A*) − EN(*B*)| and show a strongly ionic character, though the anion substructure has a covalent character following the octet rule (Schäfer *et al.*, 1973 ▸). Mainly (but not exclusively), the *A* element is an alkali or alkaline earth metal and the *B* element is a member of the boron, carbon, nitrogen, or oxygen group. Because of their ionic character, the *B* elements often behave like elements of the next higher group of the periodic table, which are isoelectronic to *B* ^−^. A typical member of the Zintl phases is NaSi with Δ EN(Na, Si) = 0.97.

The Δ EN(Si, *R*) of disilicides ranges from 0.1 to 0.5 for actinide *R* elements, from 0.6 to 0.7 for lanthanides, and from 0.8 to 0.9 for alkaline earth metals, see Fig. 2 ▸(*f*). Here, the alkaline earth metals have the highest electronegativity differences, and should therefore have the most strongly ionic character among the *R*Si_2_ and *R*_2_ *T*Si_3_ compounds. Literature confirms the ionic character only for the following compounds: EuSi_2_ (Evers *et al.*, 1977*a* ▸), Ca_2_AgSi_3_ (Cardoso Gil *et al.*, 1999 ▸), Ba_2_AgSi_3_ (Cardoso Gil *et al.*, 1999 ▸) and Eu_2_AgSi_3_ (Cardoso Gil *et al.*, 1999 ▸). The *R* elements of this group are only alkaline earth metals, Yb, and Eu, confirming the more strongly ionic character for compounds with divalent *R*.

The *R*Si_2_ and *R*_2_ *T*Si_3_ compounds with divalent *R* element can form with structure type EuGe_2_, as recent theoretical or high-pressure studies show (Evers *et al.*, 1977*b* ▸; Bordet *et al.*, 2000 ▸; Brutti *et al.*, 2006 ▸; Eisenmann *et al.*, 1970 ▸; Evers, 1979 ▸; Gemming & Seifert, 2003 ▸; Gemming *et al.*, 2006 ▸; Enyashin & Gemming, 2007 ▸; Flores-Livas *et al.*, 2011 ▸). However, those reports are outside the scope of the present article focusing on experimental reports at standard conditions. The EuGe_2_ type is a strongly perturbed version of the AlB_2_ type and resembles the structure of black phosphorus. Hence, these compounds are good candidates for Zintl phases. However, the other *R*_2_ *T*Si_3_ compounds also show polyanionic rings. For these cases, the charge of the ring is not compensated by charged ions, but by the electron gas.

#### 3.6.5. Hückel arenes   

In the next paragraph, we need to consider one complete [Si_6_] ring, see Fig. 14 ▸, thus, we discuss the corresponding sum formulas *R*_4_Si_8_ or *R*_4_ *T*_2_Si_6_, respectively. The 34 valence electron version accords with the Hückel arene description of Ba_4_Li_2_Si_6_ (von Schnering *et al.*, 1996 ▸; Cardoso Gil *et al.*, 1999 ▸). Hückel arenes are aromatic compounds that gain extra stability if 4*n* + 2 (*n* = 0, 1, 2, …) π electrons are present within a ring system and the π system is half-filled (for the present case *n* = 1) (Holleman & Wiberg, 2007 ▸). Of the 34 electrons of Ba_4_Li_2_Si_6_, 12 electrons form σ bonds and 12 further ones form coordinative bonds to the *T* element, thus, 10 π-electrons are left (von Schnering *et al.*, 1996 ▸). We conclude that the stability argument of Hückel arenes would only be valid for compounds with 24 + (4*n* + 2) = 26, 30, 34, … electrons and isolated Si hexagons induced by Si/*T* ordering. Equating the electron requirement with the formal electron contributions according to the sum formula, results in the following equation: 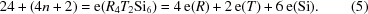
The dependence of the aromatic character on the choice of the *T* element becomes evident, because the Si sublattice yields the 24 electrons required for the σ bonds and the coordinative bonds: 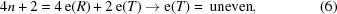
otherwise the electron system is anti-aromatic [e(*T*) is even].

Table 7 ▸ compares the amounts of AlB_2_-like *R*_2_ *T*Si_3_ compounds with an electronic Hückel configuration and ordered structures. We excluded the non-stoichiometric disilicides with and without ordered vacancies, as no isolated [Si_6_] rings are present within them. If *T* is uneven and thus the Hückel rule is fulfilled, then ordered structures are more probable than disordered ones (25% and 20%, respectively). Additionally, if the Hückel rule is broken (*T* is even), then ordered structures are less probable than disordered ones (18% and 37%, respectively). This finding could also be the reason why the *R*_2_NiSi_3_ compounds have not been reported with ordered Si/*T* atoms as they do not fulfill the Hückel rule. Moreover, this could also explain the ordered structure of Ba_2_LiSi_3_, which also has a *T* element with an uneven number of electrons. In conclusion, if the Hückel rule is formally fulfilled [e(*T*) uneven], then the formation of ordered structures is more probable, stabilized by isolated, aromatic [Si_6_] rings.

## 4. Correlations   

The following section presents and discusses results of a correlation analysis between the different properties of *R*Si_2_ and *R*_2_ *T*Si_3_ compounds that were introduced in the preceding section. Additionally, we considered the degree of ordering *n* and the crystallinity of the sample. The results for the correlations with the lattice parameters *a* and *c*, the ratio *c*/*a*, and the shortest Si—*T* distance *d* are highly redundant as these parameters are related to each other. Therefore, we focused on the ratio *c*/*a* and the shortest Si—*T* distance *d* and discussed the lattice parameters only if they gave additional information.

Some parameters did not reveal any information. For instance, the crystallinity of the sample (single crystal, crystal, ceramic, powder, thin film) did not correlate with any other property analyzed within this paper, although an absence of ordering was expected if the crystallite size was of the order of magnitude of the unit-cell parameters. Additionally, we expected correlations with the lattice parameters induced by strain within epitaxially grown thin films, which we also could not verify. Another example is the range of ordering *n*, which did not reveal correlations (except for the thermal treatment and the electronic basics of the Hückel arenes). Here, the biggest challenge is that too many data points occupy the same plot point in the discrete scale of *n*. Hence, we did not discuss these parameters separately.

Influences of the crystal lattice on other properties are included in the box plots, see Figs. 3 ▸ and 18 ▸. Additionally, the lattice type is reflected by the symbol of the following correlation plots and thus is always discussed simultaneously.

The upcoming graphics and their interpretations will be highly complex. Some general remarks are noted in §2.4.3. To facilitate the entry, we will discuss the first example at a higher level of detail than the other ones.

### 4.1. Correlations with the shortest Si—*T* distance *d*   

#### 4.1.1. Correlation of the shortest Si—*T* distance *d* with lattice parameters *a* and *c*   

The definition of the shortest Si—*T* bonds in equation (1) is based on the *a* parameter for AlB_2_-like compounds. Therefore, the correlation plot between these two properties in Fig. 4 ▸ shows a perfect line for AlB_2_-like compounds (marked by shapes: hexagon and open star). However, the ThSi_2_-like compounds (diamond and elongated diamond) deviate widely, especially for the disilicides, highlighted with purple markers and the lines labeled with ‘Si lan’ and ‘Si act’ in the left subplot. As we mentioned previously, the *a* parameter of ThSi_2_-like compounds is determined by similar local symmetries as the one of AlB_2_-like compounds. Thus, it yields *d* ≈ *a*/(3)^1/2^ approximately. The tetragonal lattice allows a distortion of the trigonal planar coordination, including varying bonding angles. Most of the compounds with ThSi_2_- and GdSi_2_-like lattice are located above the regression line of AlB_2_-like compounds, implying angles > 120° between the intrachain bonds. The largest angles arise for SrSi_2_ (colors: purple in the left subplot and purple/gray in the right subplot at *d* ≈ 2.31 Å and *a* ≈ 4.4 Å), LaSi_2_ (left: purple, right: dark blue, *d* ≈ 2.30 Å, *a* ≈ 4.3 Å), and EuSi_2_ (left: purple, right: bright blue, *d* ≈ 2.25 Å, *a* ≈ 4.3 Å), which are all disilicides without *T* element. In contrast, USi_2_ (left: purple, right: orange, *d* ≈ 2.35 Å, *a* ≈ 3.9 Å) and U_2_CuSi_3_ (left: bright blue, right: orange, *d* ≈ 2.32 Å, *a* ≈ 3.95 Å) as well as several Th (right: yellow) compounds possess intrachain bonding angles < 120°. The determination of the smallest distance *d* for the ThSi_2_-like compounds causes a slight error to the exact value as we use an approximation, see §3.2. The smallest distance *d* for ThSi_2_-like compounds is 2.20 Å, whereas AlB_2_-like compounds can exhibit smaller values down to 2.11 Å. As the ThSi_2_-like compounds incorporate the larger *R* elements, the lattice parameters are enlarged and therefore also the Si—*T* distances. For AlB_2_-like *R*_2_ *T*Si_3_ compounds the Si/*T* distances increase upon the replacement of Si by a larger *T* element.

**Figure 5:**
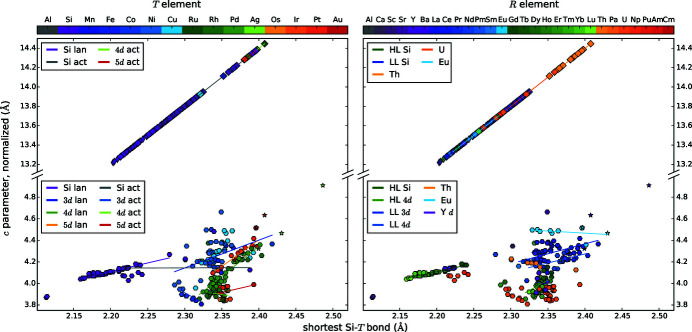
Correlations between the shortest Si—*T* distance *d* and the lattice parameter *c*. For ThSi_2_-like compounds *c* ∝ *d* by definition. The *d* value of AlB_2_-like compounds is separated in *R*Si_2_ compounds < 2.25 Å and *R*_2_ *T*Si_3_ compounds > 2.28 Å.

For ThSi_2_-like compounds, the definition of *d* is based on the lattice parameter *c*, therefore *d* depends linearly on the *c* parameter for tetragonal compounds, see Fig. 5 ▸. Within the lanthanide disilicides, the lanthanide contraction causes increasing distances *d* and lattice parameters *c* with decreasing atomic number of the *R* element for all lattice types. The *c* parameter of the AlB_2_-like disilicides is determined by the *R* radius and has a very narrow range between 4.02 Å and 4.19 Å. For AlB_2_-like *R*_2_ *T*Si_3_ compounds, the *R* element determines again the *c* parameter, whereas the period of the *T* element mainly influences the distance *d* and thus the *a* parameter. For AlB_2_-like lattices, the *c* parameters of Th compounds are comparable to those of LL (light lanthanides) compounds. In contrast, other actinides cause *c* parameters lower than those of LL and even of HL (heavy lanthanides) compounds. All *R* = Eu compounds have an almost identical *c* parameter of ≈ 4.6 Å.

#### 4.1.2. Correlation of the shortest Si—*T* distance *d* with the radius of *R*   

As given below (§A.3), the lanthanide contraction clearly influences the bond distances within the *R*Si_2_ and *R*_2_ *T*Si_3_ compounds. With increasing radius *r*_*R*_ the parameters *a* and *c* are increasing, thus the shortest Si/*T* distance *d* is also increasing, see Figs. 2 ▸(*a*), 2 ▸(*b*) and 2 ▸(*d*). In contrast, the *c*/*a* ratio is almost constant for a fixed *T* and variable *R* element (no color change), meaning that *a*, *c* and *d* are increasing at approximately the same rate, see Fig. 2 ▸(*c*).

Section A.3 also highlights the special properties of Eu and Yb compounds because of their electron configuration is composed of two *s* electrons, the half or completely filled 4*f* shell and stable, lower lying shells. As a consequence of this configuration, the metallic radii of these two elements are about 10% higher than the radii of their neighbors in the periodic table. This radius anomaly of Eu and Yb is particularly well visible as a jump in the *c* parameter and consequently also in the distance *d* and the *c*/*a* ratio.

Figs. 19 ▸(*a*) and 19 ▸(*b*) show the lattice parameters and *c*/*a* ratio for hexagonal *R*Si_2_ and *R*_2_ *T*Si_3_ compounds. It is evident that the lattice parameters change almost linearly with the radius of the *R* element. Comparing the standard errors of the indicated regression lines shows that the values for light lanthanides are generally higher than for heavy lanthanides (except for *R*_2_RhSi_3_). If we consider LL and HL separately, the trend of the lattice parameters follows different slopes, mainly steeper for compounds with HL elements. The *R* elements Y and Gd mark the transition point.

#### 4.1.3. Correlation of the shortest Si—*T* distance *d* with the radius of *T*   

The radii of the *T* elements within one period are nearly identical for the Fe, Co, and Ni group; elements of higher or lower groups exhibit larger radii. These elements not only resemble each other in radius but also in other chemical properties (Riedel & Janiak, 2011 ▸). This trend is reflected in the *a* parameter and the distance *d*, see Figs. 2 ▸(*a*) and 2 ▸(*d*).

Both, the incorporation of a *T* element into an *R*Si_2_ compound and the variation of the *R* element in *R*Si_2_, affect the dimensions of the Si sublattice, see Fig. 2 ▸(*a*). The *R* replacement causes changes of the *a* parameter on the order of 5% for the hexagonal disilicides with trivalent *R* (minimum for LuSi_2_ at 3.75 Å and maximum for NdSi_2_ at 3.95 Å). The incorporation of a *T* element (from *R*Si_2_ to *R*_2_ *T*Si_3_) has a larger effect on the lattice of an *R*Si_2_ compound than the replacement of an *R* element with another one, as the *T* element affects the Si sublattice more directly. The *R* replacement in *R*Si_2_ causes changes of the *a* parameter in the range of 3.7 Å to 3.85 Å, see Fig. 2 ▸(*a*), purple to dark blue. In contrast, the incorporation of a *T* element has a much stronger effect, for instance the Si sublattice of the compound LuSi_2_ enlarges by 7% from 3.75 Å to 4.03 Å upon Si substitution by Pd. Additionally, we compared the trend of the Si—*T* distances within the hexagonal disilicides with trivalent *R* and their ternary counterparts. The comparison reveals a weaker influence of the *R* element onto the structure of ternary compounds, which amounts to changes of about 2% (minimum for Lu_2_PdSi_3_ at 4.03 Å and maximum for Nd_2_PdSi_3_ at 4.10 Å). Hence, for larger transition metals the influences of the *R* element become less pronounced.

The subfigures of Fig. 20 ▸ on their own give an overview of the influence of *r*_*R*_ and additionally allow the analysis of the incorporation of a *T* element. Compared to Figs. 4 ▸ and 5 ▸, we receive the following additional information. The difference in slope of the *c* and *a* parameters for AlB_2_-like compounds strongly increases by the incorporation of a *T* element. For the disilicides with heavy lanthanides, the slopes are almost identical with 1.78 for the *a* and 1.74 for the *c* parameter. In contrast, for *R*_2_PtSi_3_ the slope of *a* is 0.52 and for *c* is 1.46. In the cases of Rh and Pd, the *a* parameter is even larger than *c*. Due to the incorporation of the *T* element, some of the strong covalent Si=Si bonds are replaced by weaker Si—*T* bonds. The weakened bonds elongate (*a* increases), allowing the *R* atom to sink deeper into the hexagons (*c* decreases). Mayer & Felner (1973*b* ▸) explained this phenomenon for *R*Ni_*x*_Si_2−*x*_ with purely electronic influences. As we will show in §4.5.4, the determining factor in the present case is the radius of the *T* element.

As we only found three tetragonal *R*_2_ *T*Si_3_ compounds with a lanthanide *R* element in the literature [Er_2_CuSi_3_: Raman (1967 ▸); Nd_2_AgSi_3_: Mayer & Felner (1973*b* ▸); La_2_AlSi_3_: Raman & Steinfink (1967 ▸)], a similar comparison of the influence of the *T* element onto the lattice is statistically not meaningful.

#### 4.1.4. Correlation of the shortest Si—*T* distance *d* with the ratio of lattice parameters *c*/*a*   

**Figure 6:**
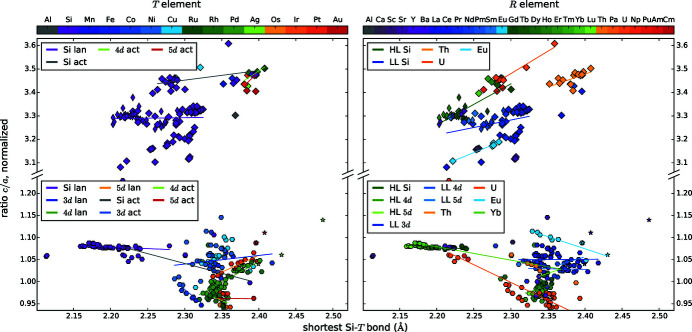
Correlations between the shortest Si—*T* distance *d* and the ratio *c*/*a*. AlB_2_-like disilicides have an almost constant *c*/*a* ≈ 1.08 like the prototype AlB_2_. The large actinide atoms elongate the weaker bonds along *c* in ThSi_2_-like compounds, and thus *c*/*a* increases.

The *c*/*a* ratio of the AlB_2_-like, lanthanide disilicides is almost constant with a value of 1.08, which is similar to the ratio in the prototype AlB_2_, see Fig. 6 ▸. However, the distance *d* is increasing with decreasing atomic number of the *R* element, and thus with increasing radius. Considering each period of *T* elements separately for hexagonal lanthanide *R*_2_ *T*Si_3_ compounds, then the ratio *c*/*a* increases with increasing distance *d*. Additionally, the group of ‘3d lan’ exhibits the highest *c*/*a* ratio at lowest *d* distances, whereas the 5d lanthanides exhibit the lowest *c*/*a* ratio at highest *d* distances. This accords with a more strongly elongated *a* parameter for larger *T* atoms, inducing a larger distance *d*. The trend of the ‘4d lan’ is very similar to the ‘5d lan’. This indicates that the steric behavior is mainly determined by the radial extension of the valence electron shell and not by finer details of the electronic structure.

For ThSi_2_-like compounds, the differentiation between different *R* elements accentuates linear dependencies. However, the LL compounds deviate strongly around their regression line. The Si—*T* distances mainly lie below 2.32 Å for ThSi_2_-type disilicides (exceptions: ThSi_2_ and USi_2_), and below 2.25 Å for hexagonal disilicides (exceptions: ThSi_2_). The huge actinides enlarge the *a* direction and thus also the distance *d*. Nevertheless, the *c*/*a* ratio hardly changes, thus, the change of *a* and *c* need to be very similar, pointing to an isotropic effect. For AlB_2_-like compounds, the *c* direction is characterized by weak van der Waals forces, thus *c* is easily stretched by the *R* atoms. In ThSi_2_-like compounds, the slightly longer intrachain bonds along *c* are also the weaker bonds, that can be stretched more easily (Mayer & Felner, 1973*b* ▸).

#### 4.1.5. Correlation of the shortest Si—*T* distance *d* with the ratio of atomic radii *q*_rad_   

**Figure 7:**
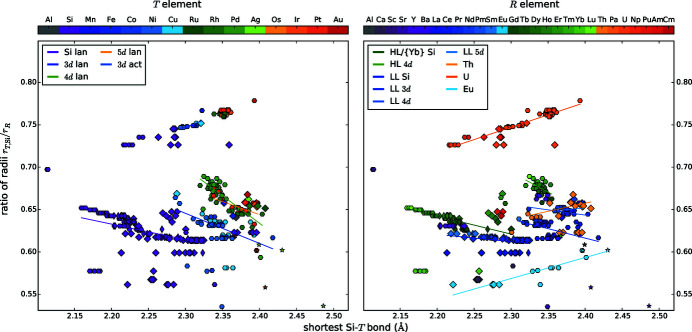
Correlations between the shortest Si—*T* distance *d* and the ratio of radii *r*_*T*, Si_/*r*_*R*_. Using one regression line for HL and LL compounds the *R* elements gives an equivalent trend, but using separate lines shows a transition in slope at *R* = Gd and Y.

Comparing the ratio of atomic radii *q*_rad_ = (*r*_*T*,Si_)/*r*_*R*_ with the shortest Si—*T* bonds *d* (Fig. 7 ▸), almost all lanthanide disilicides form a line to the same degree valid for LL and HL compounds. However, evaluating *R*Si_2_ compounds with LL and HL separately, the different slopes of these two groups become visible. The slope changes at the elements Gd and Y, which mark the transition between LL and HL. The half-filled *f* shell of Gd causes this discontinuity, which is generally called gadolinium break and influences numerous properties such as density, melting point, and ionization energies (Laing, 2009 ▸). This effect is also slightly visible in the trend of radii of the *R* elements, see Fig. 13 ▸. Outliers are the Eu and Yb compounds, which reflects both the difference of the valence shell occupation and the concomitant discontinuity of the metallic radii. Also, the La disilicides do not follow the overall trend, as the *f* shell of La is not occupied and therefore the radius of La^3+^ is slightly higher than those of the other trivalent lanthanide ions. The regression lines for *R* = Eu, Th compounds have almost identical slopes.

#### 4.1.6. Correlation of the shortest Si—*T* distance *d* and the density   

**Figure 8:**
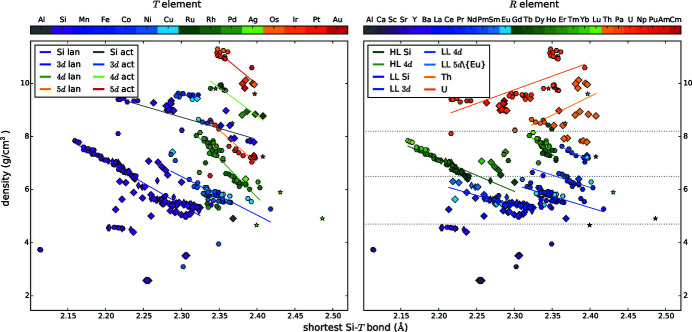
Correlations between the density and the shortest Si—*T* distance *d*. The *R* element strongly determines the density. The density linearly depends on the chosen *R* and *T* elements. With increasing atomic number of the *R* elements, *r*_*R*_ increases and thus *d* decreases and simultaneously the density decreases. With increasing atomic number of the *T* elements, *r*_*T*_ increases and thus *d* increases and simultaneously the density increases.

The density and the shortest Si—*T* distance show clear linear dependencies with respect to the *T* element as well as to the *R* element, Fig. 8 ▸. Considering the periods of the *T* elements separately, the shortest Si—*T* distance increases with increasing density. The lowest densities and shortest *d* bonds are present for the lanthanide disilicides, followed by 3d, 4d and 5d lanthanides. The actinide compounds succeed with the same sequence of *T* classes. The trends of all groups exhibit a similar slope, except for the actinide compounds with a slightly flatter slope. Because of the lanthanide contraction, the density decreases with increasing atomic number, see *e.g.* HLSi.

For the U compounds, the *T* groups do not form a line, but more a triangle. This is justified, as the masses of *T* elements of different periods differ significantly, but 4d and 5d elements have similar radii and considerable higher radii than 3d elements (lanthanide contraction, see §A.3). Thus, the densities of 3d and 4d compounds are comparable (with 9.52 g cm^−3^ and 9.82 g cm^−3^ in average, respectively), whereas the distance *d* is larger for 4d compounds (2.35 Å instead of 2.31 Å). In contrast, 4d and 5d compounds have nearly the same *d* of 2.35 Å, but the density of the 5d compounds is with 11.12 g cm^−3^ larger. This also holds for the Th_2_ *T*Si_3_ compounds.

As the density strongly depends on the *R* element, it characterizes the composition. Densities below ρ < 4.8 g cm^−3^ only appear for compounds including *R* = Al, Ca, Sc, Sr, Y, Ba. In the next higher group, up to ρ < 6.4 cm^−3^, mainly LL compounds arise, exceptions are Y compounds with 4d elements. The succeeding group (ρ < 8.3 g cm^−3^) comprises mainly HL compounds, but also Y with 5d elements, LL with 5d elements, and some few LL with 4d elements. The group with the highest density contains the actinides and HL compounds with 5d elements.

Generally, with increasing atomic number of the *R* element, the distance *d* decreases and the density increases.

#### 4.1.7. Correlation of the shortest Si—*T* distance *d* with the atomic packing factor   

**Figure 9:**
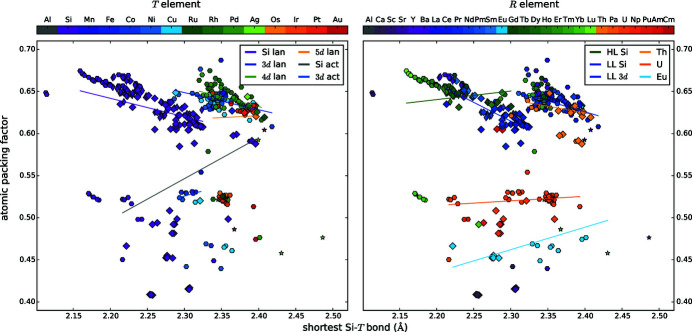
Correlations between the atomic packing factor and the shortest Si—*T* distance *d*. The apf is mainly determined by the *R* element, visible in the almost horizontal lines for the *R* elements Th, U and Eu.

The *R*Si_2_ compounds form a distinct group in the correlation plot of distance *d* and atomic packing factor, see Fig. 9 ▸. Again, these disilicides form a nearly perfect line. A second group is formed by *R*_2_ *T*Si_3_ compounds with *R* being Th or a lanthanide (except Eu, Yb). This group forms a broad cluster with *d* ∈ [2.30, 2.45] and apf ∈ [0.62, 0.7]. A last group is formed by the *R*_2_ *T*Si_3_ compounds containing actinides except Th. Compared with the two other groups, the apf is significantly lower (below 0.55, instead of above 0.6). In particular the compounds containing noble metals exhibit unusually low apf, probably caused by uncertainties in determining a radius of the noble metal (see Table 8 ▸) and therefore inaccurate apf.

The apf is mainly influenced by the incorporated *R* element, as the apf of compounds with the same *R* is almost identical (horizontal regression lines for Th, Eu, U).

### 4.2. Correlations with the ratio of parameters *c*/*a*   

#### .4.2.1. Correlation of the ratio of parameters *c*/*a* with the lattice parameter *c*   

**Figure 10:**
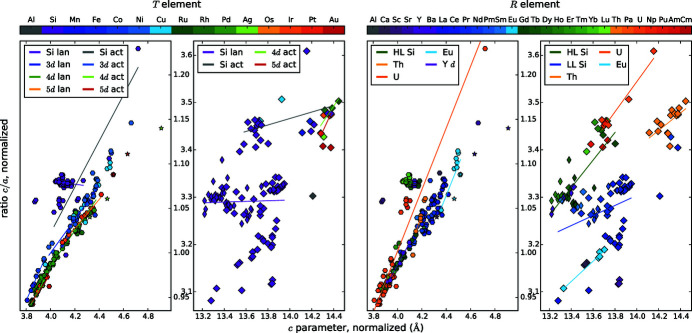
Correlations between *c*/*a* ratio and the lattice parameter *c*. The ratio *c*/*a* depends linearly on the *c* parameter of AlB_2_-like *R*_2_ *T*Si_3_ compounds, with stronger influences from the *R* element (generally, large *R* means large *c* and large *c*/*a*).

The *c*/*a* ratio and the *c* parameter of hexagonal *R*_2_ *T*Si_3_ compounds correlate linearly with each other, see Fig. 10 ▸. The *T* elements are distributed along the complete range, only influencing the *y*-intercept (higher period means lower intercept). However, the *R* elements have a stronger influence. For the lanthanide compounds we found the following correlation: the larger the *R* element, the larger both the *c* parameter and the *c*/*a* ratio. The AlB_2_-like actinide compounds behave similarly to the HL compounds, except that Th compounds have higher *c*/*a* ratios and *c* parameters. Most disilicides (except for some that contain actinide *R* elements) cluster separately at *c* ≈ 4.1 Å and *c*/*a* ≈ 1.08.

The values for ThSi_2_-like compounds are widely spread. The *c*/*a* ratio is very low (*c*/*a* ≈ 3.15 Å) for the large Eu atom. In contrast, U and Th compounds have the highest ratios (*c*/*a* up to 3.6). This difference may be related to a stronger anisotropy of the U and Th atoms compared to the outer spherical *s* shell of Eu^+II^ (Frontzek, 2009 ▸).

#### 4.2.2. Correlation between the ratio of parameters *c*/*a* and the radii *r*_*R*_   

The lanthanide contraction (see §A.3) influences the *c*/*a* ratio indirectly by affecting both lattice parameters. Fig. 2 ▸(*c*) shows that the *c*/*a* ratio is almost constant if the *T* element is fixed and the *R* element varies within the lanthanides. This constancy means that both *a* and *c* change approximately at the same rate, see also Fig. 19 ▸(*a*). The *c*/*a* ratio also reflects the radius anomaly of Eu and Yb. The abrupt increase of the radius is particularly well visible as a jump in the *c* parameter and also in the *c*/*a* ratio, but not in the *a* parameter, see Figs. 2 ▸(*a*)–2 ▸(*c*). This could originate from the different interatomic potentials for in- and out-of-plane directions. While the in-plane potential is defined by covalent bonds, the out-of-plane potential is characterized by van der Waals forces. Hence, the equilibrium of the latter is less pronounced and the distances are more flexible.

### 4.3. Correlations with ratio of radii *q*_rad_ = *r*_*T*,Si_/*r*_*R*_   

#### 4.3.1. Correlation between symmetry and *r*_*T*_   

Mayer & Felner (1973*a* ▸) examined the influence of the *T* element size on the symmetry of the corresponding Eu_2_ *T*Si_3_ compound. They used the 3d elements Fe, Co, Ni, Cu, as well as 4d Ag, and 5d Au for their synthesis. They discovered that the samples Eu_2_CuSi_3_ and Eu_2_AgSi_3_ consisted of an AlB_2_ single phase whereas Eu_2_CoSi_3_, Eu_2_NiSi_3_, and Eu_2_AuSi_3_ had additional phases and Eu_2_FeSi_3_ did not form in the AlB_2_ phase at all. They stated that the radii of the *T* elements increase as follows *r*(Co) < *r*(Ni) < *r*(Cu) < *r*(Si) < *r*(Ag) < *r*(Au) and concluded that small *T* atoms are favored in the Eu_2_ *T*Si_3_ compounds because of the reduced space originating from the large Eu^+II^ ions. We tried to reconstruct this reasoning with our data. Unfortunately, Mayer *et al.* (1967 ▸) neither defined which kind of radii they used for their assessments nor the values themselves. The radii are not calculable from the lattice parameters and interatomic distances in the same manner as they did in Mayer *et al.* (1967 ▸) for disilicides, see also Section 3. Furthermore the data are not comparable with the ones used in the present work.

Following our previous considerations and using the twelvefold coordinated metallic radii, Cu and Ag deviate about 14% and 29% from the Si radius, respectively, see Table 8 ▸. The deviations even increase when we consider an extrapolated radius for twelvefold coordinated, monovalent Cu and Ag (23% and 50%, respectively). Using the extrapolated, threefold coordinated, monovalent radius for Ag results in a very similar value to Si (only 2% deviation), but for Cu the radii are still very different (39% deviation). As copper prefers the divalent over the monovalent state, we also compared the extrapolated, divalent, threefold coordinated radius, but with even worse results (deviation of 44%). We could not apply these considerations for Au, as the list of possible radii is too incomplete for an extrapolation. In summary, we cannot confirm the deduction by Mayer & Felner (1973*a* ▸) that Eu_2_CuSi_3_ and Eu_2_AgSi_3_ form more easily than other Eu_2_ *T*Si_3_ compounds due to allegedly small radii. Additionally, our findings lead to the assumption that large *T* elements (outside of a 15% range) can be incorporated, if the Si sublattice is already expanded by large *R* elements.

#### 4.3.2. Correlation of the ratio of radii *q*_rad_ with the symmetry   

Mayer *et al.* (1967 ▸) analyzed lanthanide disilicides and discovered that AlB_2_-type structures form above *q*_rad_ = 0.579 (hereafter limit 1), whereas ThSi_2_-type structures form below this limit. The underlying interrelations and conversions were not given. By using the following equations for AlB_2_-like compounds, we receive the same values for the radii: *d*_h_(*R*, *R*) = *a* and *d*_h_(*R*, Si) = (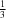 *a* ^2^ + ¼ *c* ^2^)^1/2^ as well as *r*_*R*_ = ½*d*_h_(*R*, *R*) and *r*_Si_ = *d*_h_(*R*, Si) − *r*_*R*_. Besides, they did not publish values for their tetragonal compounds. The distances *d*_t_(*R*, *R*) and *d*_t_(*R*, Si) are not unique in tetragonal compounds, as the trigonal planar coordination is slightly distorted. One possible way to estimate these distances would be to calculate upper and lower limits using the *R*–*R* distances within the *a*,*b* plane and along *c* as basis: 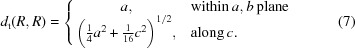
By using this redefinition of *d*(*R*, *R*) and the application of the above formula for the hexagonal lattice follows a constant ratio *q*_rad_

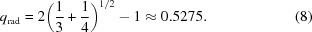
Table 9 ▸ contains the original values from Mayer *et al.* and additionally the estimated tetragonal ratios.

As outlined above, the values by Mayer *et al.* (1967 ▸) are not comparable with those used in the present article, as we used tabulated metallic radii. Thus, we determined the ratios *q*_rad_ on the basis of the tabulated values and additionally complemented the list for further lanthanide disilicides, see Table 9 ▸.

**Figure 11:**
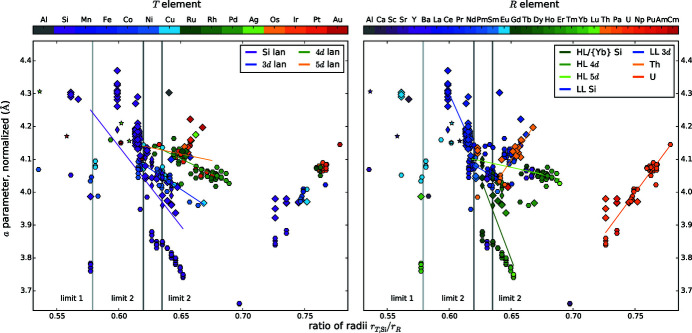
Correlations between lattice parameter *a* and ratio of radii *q*_rad_. Highlighted are the limit 1 from Mayer *et al.* (1967 ▸) and our adapted limits 2. The actinide compounds are located in the intermediate area of *q*_rad_ between HL and LL compounds, due to the size of their atomic radii.

In contrast to Mayer *et al.* (1967 ▸), we rather observe a smooth transition from tetragonal via orthorhombic to hexagonal symmetry, thus we define two approximate transition points at *q*_rad_ ≈ 0.620 and ≈ 0.635, respectively, hereafter referred to as limit 2. Hence, we cannot confirm limit 1 from Mayer *et al.* (1967 ▸) at *q*_rad_ = 0.579, because limit 1 only describes one transition in contrast to the two transitions of limit 2. Additionally, the value of limit 1 does not correspond to neither of the two values of limit 2. However, the limits 2 are only valid for the lanthanide disilicides but not for the complete range. Counterexamples are tetragonal U_2_CuSi_3_ and NpSi_2_ with very high *q*_rad_ and hexagonal Eu_2_ *T*Si_3_ with low *q*_rad_. The boxplot Fig. 3 ▸ shows the *q*_rad_ range of all *R*Si_2_ and *R*_2_ *T*Si_3_ compounds according to their lattice type. Besides the narrow range of GdSi_2_-like compounds, the *q*_rad_ of all other symmetries spans the complete range of *q*_rad_, see Table 10 ▸ and Fig. 11 ▸. Therefore, those limits only seem to apply for lanthanide disilicides and cannot be generalized.

#### 4.3.3. Correlation of the ratio of radii *q*_rad_ with the *a* parameter   

The following paragraph evaluates the correlations of the ratio of radii *q*_rad_ with the lattice parameter *a*. Fig. 11 ▸ shows that the lattice parameter *a* of lanthanide disilicides linearly decreases with increasing ratio for all structure types. This correlation is caused by a decreasing *r*_*R*_ which results in decreasing *a* as well as in increasing *q*_rad_ = *r*_Si_/*r*_*R*_. The HL disilicides (mostly hexagonal) are dominant at lower *a* and the LL (mostly tetragonal) at higher *a* values. The difference of the *a* parameter between both lattices is significant, with *a*_h_ < 3.9 Å and *a*_t_ < 4.0 Å for most lanthanide disilicides.

Surprisingly, actinide compounds have *a* values in the intermediate range, and neither at the lowest range as expected from their mass and high atomic number nor at highest range as expected due to their chemical similarity to LL. Here, the radii exert the dominant influence, whereas mass and chemical similarity play a negligible role.

With minor differences regarding the slope, increasing *q*_rad_ by decreasing *a* is also valid for the *T* element of the groups of 3d, 4d and 5d, and *R* being a lanthanide. As expected from their sequence in the periodic table, the compounds including 3d elements have the lowest *q*_rad_ and *a* values, whereas the 5d compounds exhibit the highest values. The differences in *q*_rad_ and *a* between lanthanide compounds with 4d and 3d elements is larger than between the corresponding 4d and 5d compounds, due to the very similar radii of 4d and 5d elements resulting from the lanthanide contraction, see §A.3.

For compounds with *R* = Th and U, *a* increases with increasing *q*_rad_. This does not contradict the previous assumption and the resulting grouping in HL and LL as the *R* element is fixed for the Th and U compounds. Only the *T* element affects the differences in *a* and *q*_rad_.

#### 4.3.4. Correlation of the ratio of radii *q*_rad_ with the *c* parameter   

**Figure 12:**
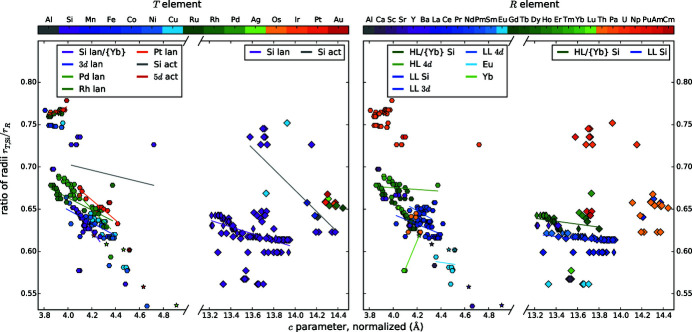
Correlations between lattice parameter *c* and ratio of radii *q*_rad_. The slope of the regression line related to different *T* elements is almost identical, but the intersect is increasing with increasing period number of the *T* element.

Plotting the *c* parameter against the ratio of radii *q*_rad_ (Fig. 12 ▸), the differentiation between actinide and lanthanide compounds is necessary again, besides the separation of ThSi_2_-like and AlB_2_-like systems. For the AlB_2_-like lanthanide systems, the *c* parameter is increasing with decreasing *q*_rad_ within every *T* group but with different intercepts. The sensitivity of the intercept on the *T* element even allows distinguishing different *T* elements of the same period, *e.g.* Rh and Pd. For the lanthanide compounds, a decreasing *r*_*R*_ causes a strongly enhanced *c* parameter, due to the weak bonds, and an increasing *q*_rad_, due to the comparably small influence from *r*_*T*_ to the ratio.

In contrast, the AlB_2_-like actinide compounds (mainly U_2_ *T*Si_3_) exhibit increasing values of *q*_rad_ with increasing *c* for the 4d and 5d groups, because *r*_*T*_ increases within the presently studied range of 4d and 5d elements. The ThSi_2_-like disilicides follow two slightly different linear trends for LL and HL, with transition at Gd and Y. Exceptions are elements with large radii *R* = Eu, La. The ratio of radii of the most ThSi_2_-like Th compounds is almost constant at *q*_rad_ ≈ 0.65. This group contains the compounds with 4d and 5d elements, which have very similar radii. Compounds with different *q*_rad_ either belong to the disilicides (without enlarging *T* element) or to 3d compounds (with small *T* element).

### 4.4. Correlations with the thermal treatment   

After comparing the *R*–*T* plots summarizing the thermal treatment Fig. 2 ▸(*h*) and the range of ordering Fig. 2 ▸(*g*), we suggested a connection between those two parameters. We created an overview of the absolute appearances of ordered structures and the application of the different thermal treatments, see Table 11 ▸. Additionally, we complemented the table with the conditional probabilities for Si/*T* ordering given a certain thermal treatment TT: 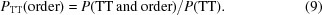
We distinguish between the Floating Zone Method (FZM), other thermal treatments (OTT, heating of the sample for more than three days at more than 450°C), and no thermal treatment (NTT). Additionally, we highlight different aspects of the compositions. Besides the evaluation of the complete list of compounds (group 1), we chose three additional groups. For group 2, we focused on the AlB_2_-like compounds and excluded the ThSi_2_-like ones, as they have not been reported with Si/*T* ordering until now. Additionally, we excluded potential vacancy ordering and thus all binary compounds (group 3). And finally, we evaluated only the disilicides with AlB_2_-like symmetry (group 4).

Table 11 ▸ reveals that the application of any thermal treatment enhances the probability of ordered structures, except for group 4. For groups 1 to 3, the probability for ordering lies above 42% for thermally treated samples and below 19% for untreated samples. Hence, the missing heat treatment of the *R*_2_NiSi_3_ samples could be the reason for missing reports regarding Si/*T* ordering. Thus the thermodynamic equilibrium structure of these compounds is very likely still undetected. Then again, the formation of ordered structures is highly favored in some other compounds so that a thermal treatment is not necessary, *e.g.* for the unintentionally grown Ba_4_Li_2_Si_6_ (Gladyshevskii, 1959 ▸; Axel *et al.*, 1968 ▸; von Schnering *et al.*, 1996 ▸).

In contrast, for group 4, the thermal treatment does not seem to benefit the formation of ordered structures. The amount of valid structure reports for group 4 is rather low compared to the other three groups discussed within this paragraph. Hence, every new report could change the statistics significantly. Furthermore, group 4 contains 22 thin films, of which the correct categorization of the thermal treatment is challenging, as shorter treatments may already be sufficient to enable vacancy ordering.

In general, the application of any thermal treatment strongly increases the probability of ordered structures. For the present data, the impact of the FZM is weaker than for OTT.

### 4.5. Correlations with electronic influences   

#### 4.5.1. Electronics and Si vacancies   

Articles about lanthanide disilicides frequently reported non-stoichiometry within these compounds, see §3.1.4. In contrast, non-stoichiometric alkaline earth disilicides have not yet been reported. We assume that these compounds have an electronic configuration, which is more favorable in comparison with the lanthanide disilicides, which try to compensate the high electron amount by non-stoichiometry. This accords with the assumption of Gorbachuk (2013 ▸) that a vec mismatch would lead to defects. The comparison of the respective valence electron amount vea listed in Table 12 ▸ confirms this theory, assuming the number of valence electrons to be e(Si) = 4, e(*L*) = 3, and e(*A*) = 2, for lanthanides *L* and alkaline earth metals *A*. The amount of valence electrons for all three non-stoichiometric lanthanide disilicides *L*Si_1.66_, *L*Si_1.75_, and *L*Si_1.8_ is closer to the alkaline earth disilicides than the *L*Si_2_. ThSi_2_-like *R*Si_1.75_ compounds would even reach this value which also militates against the *R*Si_1.8_ stoichiometry.

#### 4.5.2. Electronics from the *R* elements   

Generally, the lanthanides are assumed to be identical to each other from an electronic point of view as the electronic structure only differs in the deep *f* shells. In the following section, we will show that the different electron number of the *R* elements still influences the crystal structure.

For this reason, we want to first investigate the hypothetical Si sublattice, if it is undisturbed by *R* or *T* elements. We demand planar boundary conditions for the Si sublattice. If we removed the *R* atoms in a disilicide, the Si=Si distances would adopt the aforementioned distance 2*r*_Si_ of a conjugated π electron system and the van der Waals distance *r*_Si, vdW_ = 2.1 Å for hexagonal *c* direction. Further, if we only allowed the van der Waals bonds to stretch arbitrarily, almost all *R* elements could be incorporated in these grids [only sterical influences are considered; *r*_*R*,max, hexa_ = *d*(3/2)^1/2^ = 1.94 Å and *r*_*R*,max,tetra_ = (*d* ^2^ + ¼*b* ^2^)^1/2^ = 1.84 Å]. The hexagonal spaces do not offer enough room for the alkaline earth metals, Eu, and Yb, the tetragonal spaces are additionally too small for La and Ac.

Fig. 2 ▸(*d*) shows that the ideal distance of 2*r*_Si_ = 2.24 Å is realized approximately for GdSi_2_, and thus at the transition point between HL and LL disilicides. The LL elements with a larger radius than Gd cause an expansion of the Si sublattice, as expected for sterical reasons. In contrast, the HL elements should not have an effect on the lattice, as they are smaller than Gd and the electronics are very low. However, the lattice parameter decreases with increasing atomic number even for the HL. As a sterical effect would not cause a shrinkage but only an expansion of the sublattice, this effect is clearly of electronic nature.

For one explanation of this phenomenon, we use the principle of hard and soft acids and bases (HSAB). According to this principle, the attractiveness of silicon on an *R* element is highest, when their polarizability is similar. In the investigated structures, silicon is single or double negatively charged, which presents a high negative charge (for Si) at small radius. Therefore, if the *R* element has a high positive charge and a small radius, then the *R*—Si distances becomes shorter and more ionic. In conclusion, the packing becomes more dense as the radii of the lanthanides decrease with increasing atomic number, see Fig. 2 ▸(*i*).

#### Is a Si/T-ordering more likely if *T* has only few electrons?   



Chevalier *et al.* (1996 ▸) stated that a Si/*T* ordering is more probable, if the *T* element has only few electrons, after comparing U_2_ *T*Si_3_, *T* = Ru, Rh, Pd, compounds with each other. They found that U_2_RuSi_3_ is completely ordered, U_2_RhSi_3_ is partially ordered, and U_2_PdSi_3_ was completely disordered. We compared these findings with the complete range of *R*Si_2_ and *R*_2_ *T*Si_3_ compounds. However, we did not find this tendency for any other compound series. First, the majority of AlB_2_-like compounds was reported with at least one completely ordered structure, see Fig. 2 ▸(*g*). Thus, we cannot confirm that compounds with many electrons tend towards disordered structures. Second, the partially ordered structure type U_2_RhSi_3_ only arises for U_2_ *T*Si_3_ compounds. Thus, we cannot confirm the intermediate ordering for other *R* series. And third, although Chevalier *et al.* (1996 ▸) also discussed U_2_ *T*Si_3_ compounds with 3d and 4d *T* elements, they did not analyze them for the ordering phenomenon. Thus, we cannot confirm the theory proposed by Chevalier *et al.* (1996 ▸).

#### 4.5.4. Correlations of vec, metallic radii *r*_*T*_, and *r*_*R*_ with the lattice parameters *a* and *c*   

**Figure 19:**
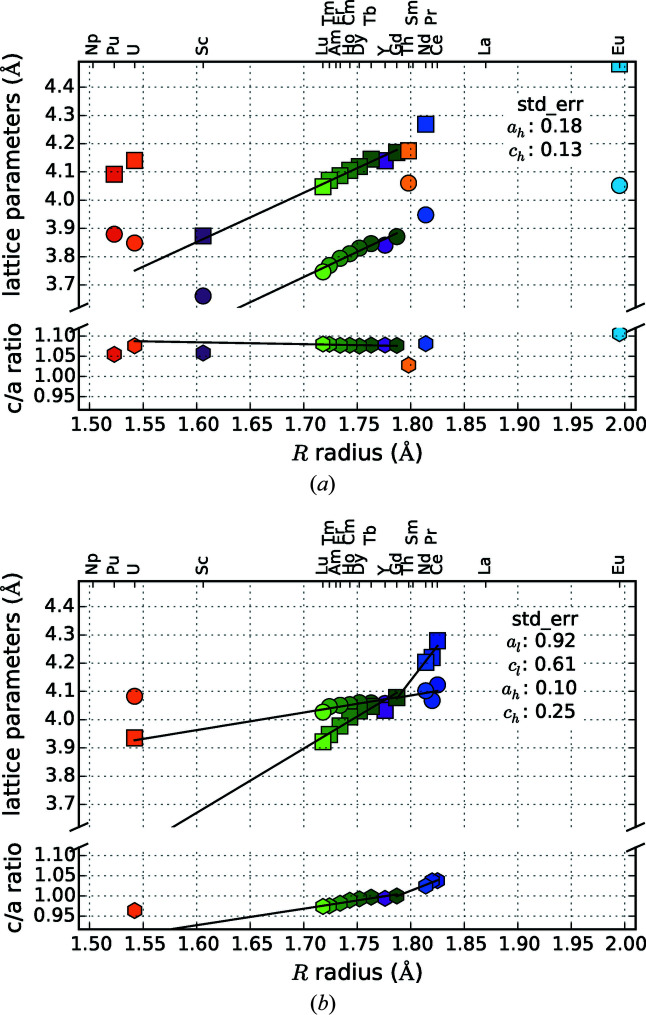
Influence of the radius of the *R* element on compounds with identical valence electron amount and constant *T* element. (*a*) hexagonal *R*Si_2_ compounds and (*b*) hexagonal *R*_2_PdSi_3_ compounds. The color changes with the atomic number of the *R* element, according to the color code from the correlation plots. Additionally, the element symbols are given at the top of the diagrams. The markers symbolize lattice parameter *a* (circle), lattice parameter *c* (square) and the ratio *c*/*a* (hexagon). The standard error is given for the indicated regression lines of the *a* and the *c* parameter, separated according light and heavy lanthanides (*h* and *l*, respectively). The color code is adapted from the correlation plots.

**Figure 20:**
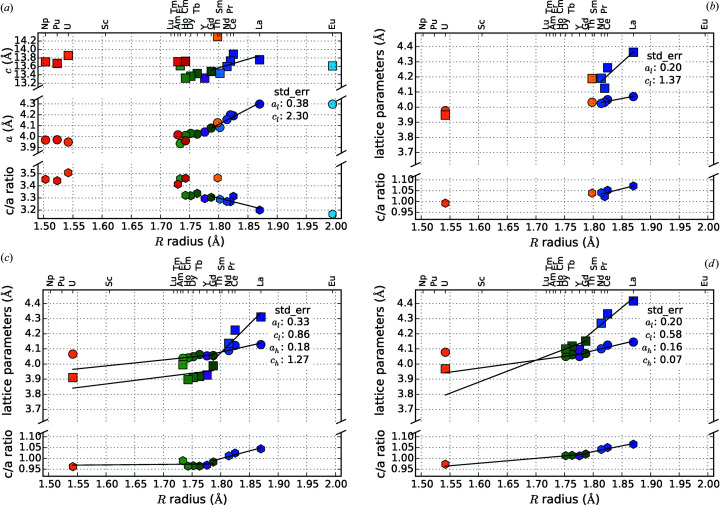
Influence of the radius of the *R* element on compounds with identical valence electron amount and constant *T* element. (*a*) tetragonal *R*Si_2_ compounds, (*b*) hexagonal *R*_2_NiSi_3_ compounds, (*c*) hexagonal *R*_2_RhSi_3_ compounds and (*d*) hexagonal *R*_2_PtSi_3_ compounds. The color changes with the atomic number of the *R* element, according to the color code from the correlation plots. Additionally, the element symbols are given at the top of the diagrams. The markers symbolize lattice parameter *a* (circle), lattice parameter *c* (square), and the ratio *c*/*a* (hexagon). The standard error are given for the indicated regression lines of the *a* and the *c* parameter, separated according light and heavy lanthanoids (l and h, respectively). The color code is adapted from the correlation plots.

Mayer *et al.* analyzed the influence of changing elements on the AlB_2_-like compounds. They concluded that those changes would affect the *c* parameter much stronger than the *a* parameter, not only for *R*Si_2_ (Mayer *et al.*, 1962 ▸), but also for *R*_2_ *T*Si_3_ compounds (Mayer & Felner, 1973*a* ▸,*b* ▸). To investigate this thesis, we plotted the *c*/*a* ratio and both lattice parameters against the radius *r*_*R*_. Fig. 19 ▸ shows the results exemplarily for hexagonal *R*Si_2_ as well as for hexagonal *R*_2_PdSi_3_ compounds and confirms that the effect of changing an element is stronger for the *c* parameter compared to the *a* parameter, as already stated by Mayer *et al.* (1962 ▸) and Mayer & Felner (1973*a* ▸,*b* ▸). Further results are listed in Appendix D[App appd]. For *R*Si_2_ compounds, both *a* and *c* increase in almost the same rate, resulting in an almost constant, slightly decreasing *c*/*a* ratio. For *R*_2_ *T*Si_3_ compounds, the influence of the *R* element on the *c* parameter is in fact larger than on the *a* parameter, resulting in an increasing *c*/*a* ratio. Therefore, we can confirm these observations of Mayer *et al.*

Mayer *et al.* also developed the theory that a lower vec would lead to weaker Si—*T* bonds compared to the covalent Si=Si bonds. These weakened bonds would elongate and thus the *c* parameter of ThSi_2_-like compounds would increase as well as the *a* parameter of AlB_2_-like compounds, thus allowing the *R* elements to sink deeper into the honeycombs and decreasing the *c* parameter (Mayer & Felner, 1973*a* ▸,*b* ▸). This theory was set up for *R*_2_ *T*_*x*_Si_2−*x*_ compounds where the varying *T* content caused the change in vec (*R* = Pr, Nd, Dy and Er as well as *T* = Fe, Co, Ni and Ag). We evaluated this theory according to its validity for our data range of *R*Si_2_ and *R*_2_ *T*Si_3_ compounds, where the change in vec is caused by varying *T* and *R* elements, which is always accompanied with changes in the radii.

As we already stated, the correct determination of the vec is challenging. However, the evaluation of a vec change may be easier, as we do not need to know the exact electron amount but only if one compound has more or less electrons than another one. The best approach to evaluate the influence of a vec change onto the lattice parameters is to compare compounds where the elements have similar radii but different valence electrons. This comparison can be realized in two ways. Either the *T* elements are fixed while the *R* elements vary or the *R* elements are fixed while the *T* elements vary. In the first approach, the *R* elements are arranged in groups so that their radii differ by a maximum of 5% and that at least one element with a different amount of valence electrons is contained. These constraints apply for *R* ^1^ = Ca, La, Eu, Yb and *R* ^2^ = Al, Sc U, Np, Pu.

**Figure 21:**
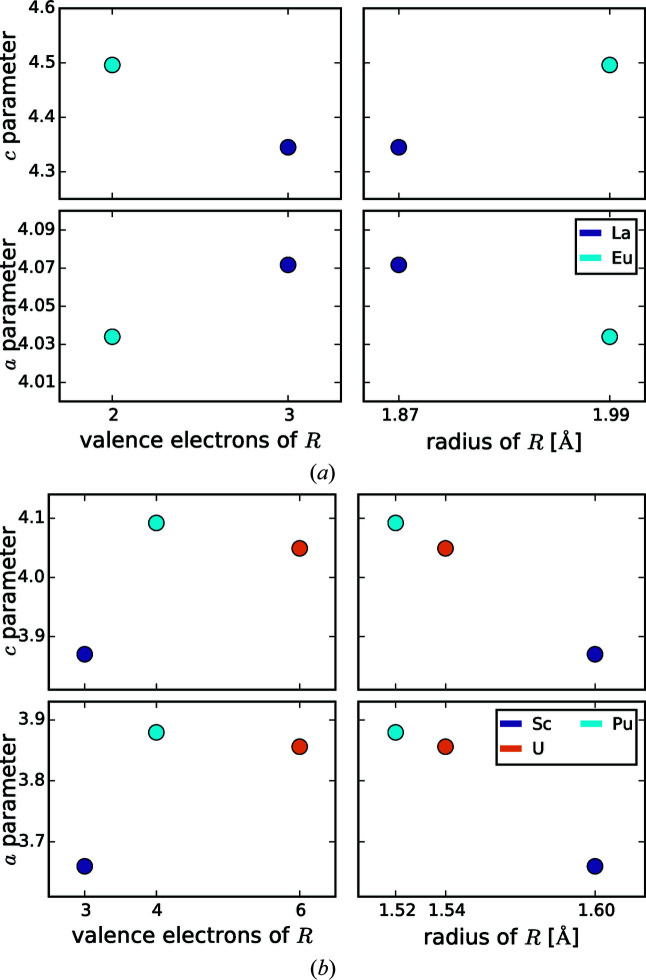
Influence of the valence electron amount on the lattice parameters of AlB_2_-like *R*Si_2_ and *R*_2_ *T*Si_3_ compounds, for *R* elements with similar radii and constant *T* element. (*a*) *R* ^*h*, 1^ = La, Eu, *T* = Ni and (*b*) *R* ^*h*, 2^ = Sc, U, Pu, *T* = Si.

For AlB_2_-like structure types, the series of *R* ^*h*, 12^NiSi_3_ compounds with *R* ^*h*, 1^ = La, Eu and of *R*Si_2_ compounds with *R* ^*h*, 2^ = Sc, U, and Pu exist. Fig. 21 ▸ shows the lattice parameters *a* and *c* in dependence of the valence electrons of the *R* element and of the radius of *R*. Both Fig. 21 ▸(*a*) and Fig. 21 ▸(*b*) for AlB_2_-like compounds do not confirm the proposed correlations. An increasing *a* parameter does not lead to a decreasing *c*, in both cases. Additionally, the *a* parameter of the Sc compound in Fig. 21 ▸(*b*) is lower than for the other two, despite its *R* element has fewer electrons. However, we could identify a clear influence of *r*_*R*_: when this radius increases, both lattice parameters decrease. We already described this correlation in §4.5.2 and assume electronic attraction as reason.

In Appendix D[App appd], we present additional plots that contradict the theory of Mayer *et al.* Thus, the first approach could not verify the validity of the theory of Mayer & Felner (1973*a* ▸,*b* ▸) about the influence of the vec onto the strength of the bonds and therefore onto the lattice parameters of all *R*Si_2_ and *R*_2_ *T*Si_3_ compounds.

The second attempt includes the substitution of the *T* element while keeping the *R* element constant. The evaluation of this approach is more challenging as an exact knowledge of the electronic state of every *T* element in every compound is mandatory. In contrast to the *R* elements, the transition metals possess a plurality of preferred valence states, which cannot be predicted easily. Therefore, we skip this approach here, and only show one example in Appendix B.2. This example does not confirm the observations by Mayer & Felner (1973*a* ▸), but rather approves the influence of the radius *r*_*T*_.

In conclusion, both approaches have shown that the influence of the vec onto the lattice is negligible compared to sterical influences. The original theory from Mayer & Felner (1973*b* ▸) for *R*_2_ *T*_*x*_Si_2−*x*_ described that a decreasing vec would weaken certain bonds and lead to the elongation of the lattice parameters *c*_t_ and *a*_h_ as well as shortening of *c*_h_. However, if the vec change is accompanied with a change in radii, the sterical effect is dominant.

## 5. Conclusion and outlook   

In this article, we presented a comprehensive review of the *R*Si_2_ and *R*_2_ *T*Si_3_ compounds relating the change of different properties due to the specific choice of *R* and *T* elements. A short overview of the interplay between the properties is given in Table 13 ▸.

The two main structural aspects of these compounds are the differentiation between AlB_2_-like and ThSi_2_-like as well as between ordered and unordered. The lattice type is mainly determined by the elemental composition of the compound, with the AlB_2_-like structures being the most dominant and thus, probably, the most flexible. Mayer *et al.* assumed that a certain lattice type would only arise in a particular range of shortest Si—*T* distances for lanthanide disilicides (Mayer *et al.*, 1962 ▸). We were able to show that these limits are not applicable for the complete set of *R*Si_2_ and *R*_2_ *T*Si_3_ compounds. The elemental combinations are additionally limited, especially concerning the *T* elements, which need to be members of the Mn to Cu groups. By applying an MO-like approach to the *R*Si_2_ and *R*_2_ *T*Si_3_ compounds for the first time, we interpret the compounds similar to complexes and give reasons why only a certain range of *T* elements appears in the *R*_2_ *T*Si_3_ compounds. For Mn compounds, the hybridization with U and Th is the main reason for the ground state. We presume that Tc and Re compounds could also be stabilized due to hybridization with U and Th and think that the respective structures should exist.

The factors for the appearance of ordered structures are more complex. First, we found that the break of the Hückel rule (4*n* + 2 electrons within a ring) strongly benefits disorder in AlB_2_-like structures. Additionally, the fulfillment of this rule favors the formation of ordered structures, but in a weaker way; probably with an additional condition that needs to be identified. In the latter case, the *T* element must possess an odd number of valence electrons. Second, the probability of ordered structures is again increased by the application of a thermal treatment, like the floating zone method or a long-time annealing of the sample. The median of temperature and time used in literature were 800°C for five days. For future investigations, we recommend the structural characterization of the *R*_2_ *T*Si_3_ crystals directly after growth and again after a thermal treatment. And third, ordered structures have only been reported for AlB_2_-like structures, up to now. We tried to find reasons that speak against ordered structures with tetragonal lattice (POTS), however this arrangement seems to be plausible. We already discussed geometrical constraints in Part I. Here, we considered the geometric bond network, resulting in two different tetragonal structure models. Additionally, we performed a Bader analysis, which excluded one model and validated the other. The analysis revealed two very different charges for the two different Wyckoff positions. This accords with the different bonding mechanisms along the *c* direction depending on the connection of Si=Si or Si—T and induces different bond lengths. We recommend reinvestigating the ternary *R*_2_ *T*Si_3_ compounds in ThSi_2_-like symmetry (*e.g.* Nd_2_AgSi_3_ and Er_2_CuSi_3_) with special regard to the POTS type of ordering.

Additionally we performed Bader analysis for other compounds and revealed that the *R* elements in metallic configurations are in fact slightly charged (*R* ^+*x*^, 1.19 < *x* < 1.33). Moreover, the charges of the *R* element in ternary, AlB_2_-like compounds exhibit significantly different values depending on their Wyckoff position. Additionally, we could show influence of the *T* element’s electronegativity and Bader volume onto the Bader charges.

According to the findings about ordering, we suggest the reinvestigation of certain compounds. Using up-to-date soft- and hardware could enable the detection of weak satellite reflections pointing at ordered structures. This concerns *T* = Ni compounds, which should be thermally treated beforehand to check the ground state. Additionally, we recommend to reinvestigate the AlB_2_-like U and Th containing compounds as those form the largest group of structures without reported ordering. Furthermore, the ThSi_2_-like *R*_2_ *T*Si_3_ compounds (Er_2_CuSi_3_ and Nd_2_AgSi_3_) should be reconsidered as the last research was performed in the 80s.

In addition to the ordering of the Si/*T* atoms and the lattice type, the length of the interatomic distances are also part of the structure, in general. The main influence onto the (normalized) lattice parameters are the radii of the *R* elements. On the one hand, the radii anomaly of Eu and Yb effects very large radii for these two elements. On the other hand, the lanthanide contraction causes the shrinkage of the atomic radii of the lanthanides with increasing atomic number. This correlation is not strictly linear, but with a kink at Gd, the so-called gadolinium break. Both are directly visible for the lattice parameters.

Additionally, we could show that the influence of the mass or the chemical alikeness play negligible roles compared to the radii, especially for the actinides. If the mass or the chemical alikeness were dominant, the actinide compounds had *a* values in the low or the high range, respectively.

Moreover, as some *T* elements exceed the steric tolerance range for replacing Si atoms, we conclude that *R* elements can enlarge the cell sufficiently to allow big *T* elements to be incorporated. In addition, the *T* elements topologically decouple the closed [Si_6_] rings in the *a*,*b* plane of ordered AlB_2_-like compounds.

Against the assumption that the shielded 4*f* electrons would hardly influence the structure, we could clearly confirm the electronic influences of the *R* element on the lattice by means of increasing and decreasing lattice parameters compared to a balanced state realized by GdSi_2_. Mayer *et al.* tried to quantify this effect using the vec (Mayer & Felner, 1973*a* ▸). They developed the theory that a lower vec would lead to weaker bonds and thus to increased *a* parameters for all lattices, increased *c* parameter for ThSi_2_-like compounds, and decreased *c* parameter for AlB_2_-like compounds (Mayer & Felner, 1973*a* ▸). This theory was set up for *R*_2_ *T*_*x*_Si_2−*x*_ compounds with varying *T* content. We evaluated this theory according to its validity for our data range of *R*Si_2_ and *R*_2_ *T*Si_3_ compounds. We checked three approaches for this evaluation, which could not confirm the transferability of this theory.

In addition, we evaluated the *R*Si_2_ and *R*_2_ *T*Si_3_ compounds according to the constraints of famous material groups. The *q*_rad_ of the silicides does not accord to the one of the Laves phases of 1.225, hence an affiliation can be excluded.For the comparison with Zintl phases, we evaluated the electronegativity difference Δ|EN|. Zintl phases typically have high Δ|EN| values. We found that only compounds with a divalent *R* and monovalent *T* element can be characterized as Zintl phases. The last material group that we used here, were the Hume-Rothery phases, where the valence electron concentration (vec) is the structure driving factor, not stoichiometry. However, the correct determination of the vec is challenging. Especially for the transition metals different articles use different approaches. Thus, a comprehensive evaluation was not possible.

Finally we evaluated the structures of the *R*Si_2_ compounds. Many of those compounds with trivalent *R* elements are, in fact, Si deficient. We showed that the overall valence electron amount (vea) of the deficient lattice is almost identical to the vea of the stoichiometric alkaline earth disilicides. This configuration seems to be more stable in comparison to the stoichiometric disilicides with *R* elements favoring the metallic state.

As the *R*_2_ *T*Si_3_ are known for their magnetic properties, the extension of this review in respect to magnetic transition temperatures, magnetic coupling, and other properties, is highly recommended.

## Figures and Tables

**Figure 22:**
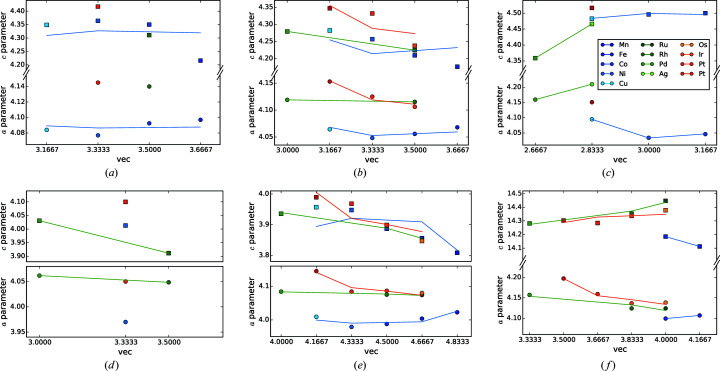
Influence of the valence electron concentration on the lattice parameters of *R*_2_ *T*Si_3_ compounds, for a constant *R* element and at least five different *T* elements. (*a*) *R* = La, hexagonal , (*b*) *R* = Ce, hexagonal, (*c*) *R* = Eu, hexagonal, (*d*) *R* = Dy, hexagonal, (*e*) *R* = U, hexagonal and (*f*) *R* = Th, tetragonal.

**Figure 23:**
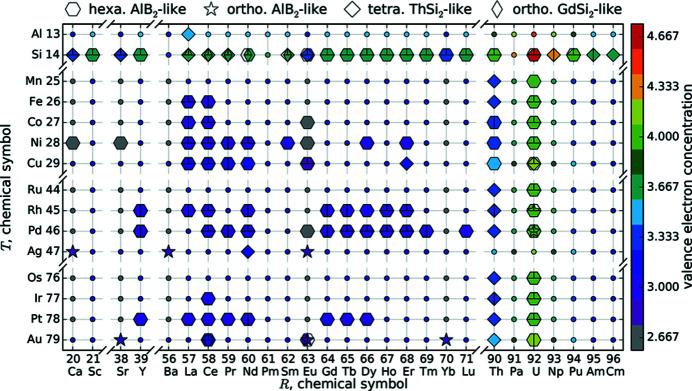
*R*–*T* diagram for the valence electron concentration vec of the *R*Si_2_ and *R*_2_ *T*Si_3_ compounds. The used markers symbolize the crystal system: hexagon — hexagonal AlB_2_-like systems, open star — orthorhombic, AlB_2_-like systems, diamond — tetragonal ThSi_2_ systems, elongated diamond — orthorhombic GdSi_2_ systems.

**Figure 18:**
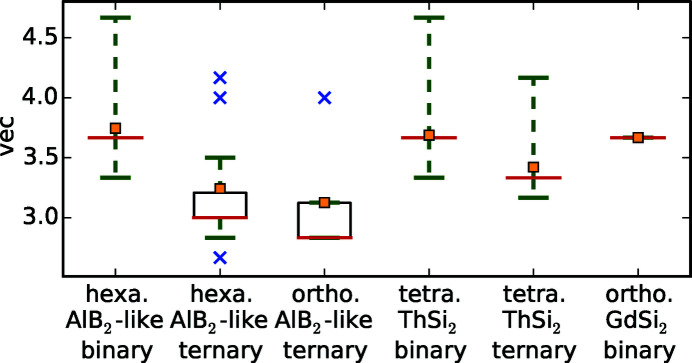
Box plot of the valence electron concentration vec. Orange square indicates average, red line indicated median, black box are limits of quartiles, green whiskers are 15th and 85th percentiles, blue crosses are outliers.

**Figure 24:**
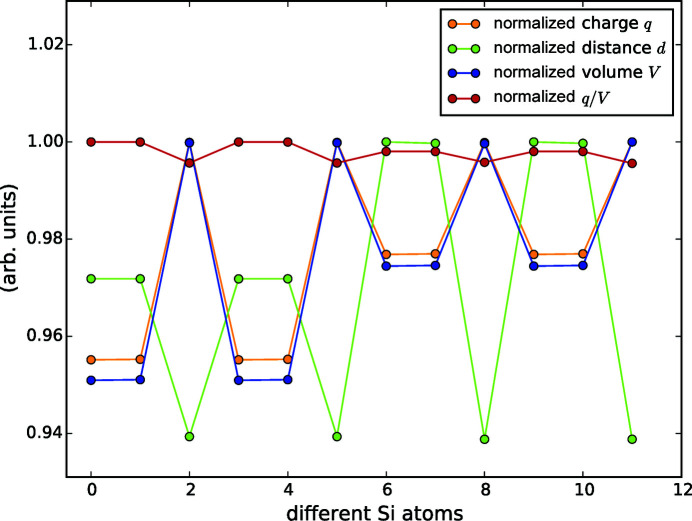
Influence of volume, charge and minimal distance (determined with the Bader analysis) onto each other.

**Figure 25:**
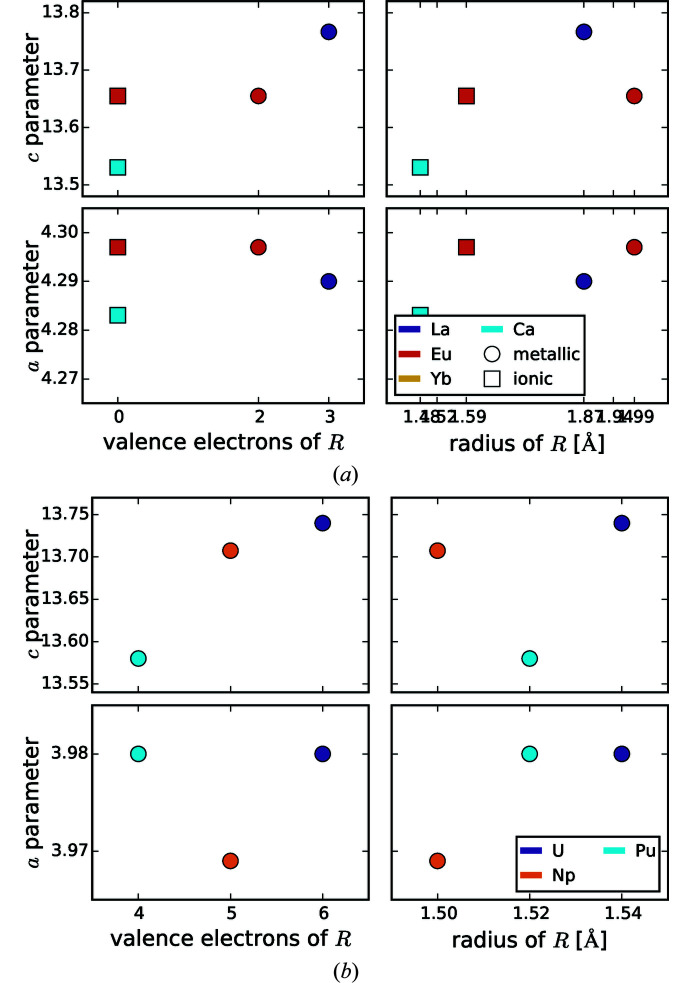
Influence of the valence electron amount on the lattice parameters of ThSi_2_-like *R*Si_2_ and *R*_2_ *T*Si_3_ compounds, for *R* elements with similar radii and constant *T* element. (*a*) *R* ^*t*,1^ = Ca, La, Eu, Yb, *T* = Si and (*b*) *R* ^*t*,2^ = U, Np, Pu, *T* = Si.

**Table 1:** Correspondence of degree of ordering *n* to the structure types introduced in Part I

	Structure types			
*n*	Hexa. AlB_2_-like	Ortho. AlB_2_-like	Tetra. ThSi_2_	Ortho. GdSi_2_
0	AlB_2_	Er_3_□Si_5_	ThSi_2_	GdSi_2_
1	Ce_2_CoSi_3_, U_2_RhSi_3_,	Ho_3_□Si_5_		
	Yb_3_□Si_5_			
2	Er_2_RhSi_3_ ( 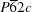 ),	Ca_2_AgSi_3_		
	Er_2_RhSi_3_, Tb_3_□Si_5_			
4	Ba_4_Li_2_Si_6_			
8	Ho_2_PdSi_3_			

**Table 2:** Ranges of shortest Si—*T* distances *d* with respect to the crystal system

	Shortest Si—*T* distance *d* (Å)		
	Mayer *et al.* (1962 ▸)	This article	
Crystal system	Disilicides	Disilicides	All
ortho. AlB_2_-like	–	–	2.35⋯2.49
AlB_2_-like	2.16⋯2.18	2.16⋯2.34	2.11⋯2.41
GdSi_2_	2.22⋯2.28	2.20⋯2.32	2.20⋯2.33
ThSi_2_	2.28⋯2.31	2.21⋯2.40	2.21⋯2.43

**Table 8:** Radii of the *T* elements as well as the relative difference of *r*_T_ and *r*_Si_ = 1.12 Å for different oxidation states and coordination numbers Radii marked with * are extrapolated values. Too few data points for the radii of monovalent Au do not allow for extrapolation.

Element	Type of radius	*r*_*T*_ (Å)	1 − *r_T_*/*r*_Si_
Mn	*M*_12_	1.37	−0.22
Fe	*M*_12_	1.26	−0.13
Co	*M*_12_	1.25	−0.12
Ni	*M*_12_	1.25	−0.11
Cu	*M*_12_	1.28	−0.14
	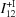	1.38*	−0.23
	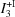	0.68*	0.39
	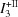	0.63*	0.44
Ru	*M*_12_	1.33	−0.18
Rh	*M*_12_	1.35	−0.20
Pd	*M*_12_	1.38	−0.23
Ag	*M*_12_	1.45	−0.29
	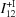	1.68*	−0.50
	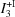	1.10*	0.02
Os	*M*_12_	1.34	−0.19
Ir	*M*_12_	1.36	−0.21
Pt	*M*_12_	1.37	−0.23
Au	*M*_12_	1.44	−0.29

**Table 3:** Density deviation 1 − ρ_*h*_/ρ_*t*_ of tetragonal and hexagonal lattices for dimorphic *R*Si_2_ and *R*_2_ *T*Si_3_ compounds

*R*	*T*	Deviation (%)
Ce	Au	1.08
U	Cu	0.32
Y	Si	0.84
Gd	Si	−1.72
Tb	Si	−1.04
Dy	Si	−1.58
Ho	Si	−1.70
Th	Si	−4.76
U	Si	−3.70
Pu	Si	−1.07

**Table 4:** Overview of the electronic contribution of the *T* element for *R*_4_ *T*_2_Si_6_ building units including one [Si_6_] ring, using the formula vea = 4 e(*R*) + 2 e(*T*) + 6 e(Si), with e(Si) = 4 The values are given for different valence electron amounts (vea) and different electronic contributions by the *R* element (2 e^−^ for the ionic state and 0 e^−^ for the metallic state). Up to now, the ionic state was only reported for divalent *R* and monovalent *T* elements, other combinations with divalent *R* are improbable.

	e(*R*) =		
vea	0	+1	+2
28	+2	+0	−2
30	+3	+1	−1
32	+4	+2	+0
34	+5	+3	+1
36	+6	+4	+2

**Table 5:** Overview of the DFT-calculated Bader charges *c* and volumes *V* for the elements representative *R*_2_Si_4_ and *R*_2_ *T*Si_3_ compounds with different symmetries The given charges and volumes are averaged over all Wyckoff positions. Additionally, the electronegativity value EN of *T* is given.

		NdSi_2_		Nd_2_AgSi_3_			Nd_2_CuSi_3_		
		Tetra	Hexa	Tetra	Hexa	Nd_2_PdSi_3_	*P*6/*mmm* (No. 191)	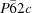 (No. 190)	Nd_2_NiSi_3_
*c*(Nd)		1.20	1.19	1.32	1.29	1.32	1.26	1.26	1.33
*c*(*T*)		–	–	−0.77	−0.85	−1.14	−0.64	−0.63	−0.76
*c*(Si)		−0.60	−0.60	−0.62	−0.58	−0.50	−0.63	−0.63	−0.55
*V*(Nd)	abs.	20.42	20.45	21.19	21.63	21.01	20.96	20.86	20.05
	rel. (%)	17.01	17.14	16.52	16.74	16.80	17.52	17.50	17.54
*V*(*T*)	abs.	–	–	22.48	23.03	23.07	17.34	17.30	17.10
	rel. (%)	–	–	17.37	17.83	18.44	14.49	14.51	14.96
*V*(Si)	abs.	19.69	19.61	21.20	20.97	20.00	20.13	20.07	19.04
	rel. (%)	16.46	16.43	16.53	16.23	15.99	16.82	16.83	16.66
EN(*T*)		–	–	1.93	1.93	2.20	1.90	1.90	1.91

**Table 6:** Si—*T* distances within POTS Nd_2_AgSi_3_

Type	Length (Å)
*d*_intra_(Si, Ag)	2.48
*d*_intra_(Si, Si)	2.38
*d*_inter_(Si, Ag)	2.49
*d*_inter_(Si, Si)	2.35

**Table 9:** Ratio of radii *q*_rad_ for *R*Si_2_ taken from Mayer *et al.* (1967 ▸) and calculated from tabulated values, with *R* being a lanthanide or Y The original data is complemented by estimations for the interatomic distances in ThSi_2_-like compounds according equation (8) (numbers in blue). Limit 1 separates orthorhombic and hexagonal compounds according to the data from Mayer *et al.* (1967 ▸). According to the data presented in this work, the additional transition to the tetragonal phase (indicated by limit 2) is highlighted.

	Mayer *et al.* (1967 ▸)				This work			
Element	*r*_*R*_ (Å)	*r*_Si_ (Å)	*r*_*R*_/*r*_Si_	Symmetry	*r*_*R*_ (Å)	*r*_Si_ (Å)	*r*_*R*_/*r*_Si_	Symmetry
La	2.135⋯2.043	1.126⋯1.078	0.528	*o*	1.870	1.120	0.599	*t*
Ce	2.095⋯2.033	1.105⋯1.073	0.528	*t*	1.825	1.120	0.614	*t*
Pr	2.085⋯2.018	1.100⋯1.064	0.528	*o*	1.820	1.120	0.615	*t*
Nd	2.085⋯2.000	1.100⋯1.055	0.528	*o*	1.814	1.120	0.617	*t*/*o*
---------------------------------------------*limit* 2---------------------------------------------								
Sm					1.802	1.120	0.622	*t*/*o*
Eu					1.995	1.120	0.561	*t*
---------------------------------------------*limit* 1---------------------------------------------								
Gd	1.934	1.120	0.579	*h*	1.787	1.120	0.627	*h*/*o*
Tb	1.922	1.114	0.580	*h*	1.763	1.120	0.635	*h*/*o*
---------------------------------------------*limit* 2---------------------------------------------								
Dy	1.915	1.107	0.578	*h*	1.752	1.120	0.639	*h*/*o*
Ho	1.900	1.103	0.581	*h*	1.743	1.120	0.643	*h*/*o*
Er	1.892	1.098	0.580	*h*	1.734	1.120	0.646	*h*
Tm	1.885	1.095	0.581	*h*	1.724	1.120	0.650	*h*
Lu	1.874	1.089	0.581	*h*	1.718	1.120	0.652	*h*
Y	1.917	1.113	0.581	*h*	1.776	1.120	0.631	*h*

**Table 10:** Limits of the ratio of radii *q*_rad_ determining the symmetry of the disilicides (for columns one and two) and for the complete data range (column three)

Symmetry	Mayer *et al.* (1967 ▸)	Applying radii from Holleman & Wiberg (2007 ▸)	Boxplots
ThSi_2_	⋯0.579	⋯0.620	0.50⋯0.80
GdSi_2_	–	0.620⋯0.635	0.60⋯0.65
AlB_2_-like	0.579⋯	0.635⋯	0.50⋯0.80
ortho. AlB_2_-like	–	–	0.53⋯0.77

**Table 11:** Overview of the number of reports on thermal treatments and the appearance of ordering in different groups of *R*Si_2_ and *R*_2_ *T*Si_3_ compounds We distinguish between the application of the Floating Zone Method (FZM), other thermal treatments (OTT, heating of the sample for more than three days at more than 450°C), and no thermal treatment (NTT). Additionally, we present the conditional probabilities of Si/*T* ordering given that a certain thermal treatment TT was applied *P*_TT_.

	FZM	OTT	NTT	Order	Disorder	*P*_FZM_(order) (%)	*P*_OTT_(order) (%)	*P*_NTT_(order) (%)
1: all compounds	12	146	277	90	345	42	42	8
2: all AlB_2_-like	12	121	141	90	184	42	51	16
3: AlB_2_-like, ternary	12	113	59	77	107	42	54	19
4: AlB_2_-like, binary	0	8	34	13	29	–	13	35

**Table 12:** Comparison of the valence electron amount vea for *R*Si_2_ compounds with different stoichiometries and *R* elements (alkaline earth *A* or lanthanide *L*) e(Si) = 4, e(*L*) = 3, e(*A*) = 2.

Composition *c*	vea(*c*)	Δ[vea(*c*), vea(*A*Si_2_)]
*A*Si_2_	10.0	0.0
*L*Si_2_	11.0	1.0
*L*Si_1.66_	9.7	0.3
*L*Si_1.75_	10.0	0.0
*L*Si_1.8_	10.2	0.2

**Table 7:** Overview of the number of AlB_2_-like *R*_2_ *T*Si_3_ compounds with Hückel configuration [e(*T*) uneven] and ordered structures

		e(*T*) uneven	e(*T*) even
		83 (45%)	101 (55%)
Order	79 (43%)	46 (25%)	33 (18%)
Disorder	105 (57%)	37 (20%)	68 (37%)

**Table 13:** Summary of the correlations between different parameters

	Distance *d*	Ratio *c*/*a*	Ratio *q*_rad_	Thermal treatment	Electronics
*a*	AlB_2_-like: proportionality; ThSi_2_-like: wide deviations; Fig. 4(*f*) ▸	*R*Si_2_: *a* ≈ 1.08 Å; *R*, *T* only slightly affect *a*; *c*/*a* strongly sensitive to *R*	‘lan Si’: linear, decr. *r*_*R*_ → decr. *a* and incr. *q*_rad_; *a* mainly influenced by *R*; Fig. 11 ▸		Geometric discussion: electronic attraction of *R* → lattice shrinkage
*c*	ThSi_2_-like: proportionality, incr. atomic number → incr. *c* and *d*; Fig. 5 ▸	Proportionality, depending on symmetry; for ‘lan Si’ at *c*/*a* = 1.08, *c* = 4.1 Å; Fig. 10 ▸	Linear dependency: *T* elements determine slope; incr. *r*_*R*_ → incr. *c* and decr. *q*_rad_; Fig. 12 ▸	–	–
*c*/*a*	actinide: incr. *c* → incr. *c*/*a*; AlB_2_-like lanthanide: 3d with high *d* and low *c*/*a*, 5d with low *d* and high *c*/*a*	–	–	–	–
*r*_*R*_, *r*_*T*_	*R*: lanthanide contraction; special role of Eu and Yb; *T*: shallow minimum for Fe, Co, Ni	–	Mayer & Felner (1973*a* ▸): similar radii of Cu, Ag and Si not confirmed	–	Stability range of *T* elements (MO theory)
*q*_rad_	Different slopes for HL and LL; Fig. 7 ▸	–	–	–	
Density	Proportionality; density strongly dependent on *R*; Fig. 8 ▸(*h*);	–	–	–	
Symmetry	–	–	Limit from Mayer *et al.* (1967 ▸) cannot be adapted for the complete data range	Probability of ordering incr. after thermal treatment	Non-stoichiometric; ThSi_2_-like: possible ordering (Bader); few electrons 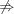 increased ordering

## Appendix A Determination of atomic radii

As we already mentioned, the determination of the radius of an element within a given structure is challenging as different influences need to be considered. Fig. 13 ▸ summarizes the radii chosen within this work and the following paragraphs explain our decisions.

### A1. The radius of Si

The Si atoms arrange in a sublattice with trigonal local symmetry and only slight puckering, in all types of structures, see Fig. 1 ▸ and Nentwich *et al.* (2020 ▸). This indicates an *sp* ^2^ hybridization of Si with a small tendency to *sp* ^3^ hybridization, which is weakened by the large *R* elements. The *sp* ^2^ hybridization is similar to the conjugated π electron system in graphene or graphite and consists in equal proportions of delocalized covalent single and covalent double bonds. The C—C bond length in graphene uniformly corresponds to the arithmetic mean of the covalent single and covalent double bonding distance. Applying this formula to the Si network results in the Si=Si bond length

Assuming densely packed atoms, the radius of Si is half the distance *d*(Si, Si), thus *r*_Si_ = 1.12 Å.

### A2. The radius of the *T* elements

The *T* elements are incorporated into the covalent Si network, therefore covalent radii were our first choice, but the tabulated data are too incomplete for a stringent use in the present discussion. Additionally, an in-plane threefold coordination would be suitable here (as imposed by the Si sublattice), but the corresponding radii are not tabulated in the standard literature (Holleman & Wiberg, 2007 ▸; Shannon, 1976 ▸; Slater, 1964 ▸) for any transition metal, neither in covalent, metallic, nor ionic state. Therefore, other approaches need to be employed.

For ionic compounds, the oxidation state of the *T* element is formally +I (Cardoso Gil *et al.*, 1999 ▸). As a corresponding radius for Ag is not listed, we extrapolated the radii of differently coordinated Ag^+I^ ions to approximate a possible radius for threefold coordination (to reflect the environment in the silicide) and for twelvefold coordination (for comparison with the metallic compounds), see Table 8 ▸ and Fig. 13 ▸. We tried the same for Au, but only one Au^+I^ radius is listed, not allowing to perform an extrapolation.

For the *T* elements in metallic compounds, we used the values for twelvefold coordinated metallic atoms (*r*_*T*, 12_) instead of the more adequate, but not completely listed threefold coordinated metallic radii. Although the coordination number is incorrect, this choice ensures a systematic (and not a random) error.

As we have already mentioned, the size of two elements determines if they can replace each other in a structure (among other factors). For Si the following elements have a good size-factor of up to +15%: Be, Fe, Co, Ni, Cu. Using a size-factor of 30%, the resulting group contains all the *T* elements found in the *R*_2_ *T*Si_3_ compounds. Thus, we conclude that *R* elements can enlarge the cell sufficiently to allow big *T* elements such as Pt to be incorporated.

Within one period, the *T* elements from the Fe, Co, and Ni groups have nearly the same metallic radius, whereas the preceding and subsequent elements have a bigger radius (see Fig. 13 ▸, green).

### A3. The radius of the *R* elements

For the *R* elements, the appropriate values for *r*_*R*_ are either the radii for twelvefold coordinated *R* ^+II^ ions for the alkaline earth metals plus Eu, Yb or the twelvefold coordinated metallic radii for the lanthanides, Sc, Y and the actinides.

One aspect determining the trend of the metallic twelvefold coordinated radii of the *R* element is the lanthanide contraction. With increasing atomic number of the *R* element within the lanthanides (see Fig. 13 ▸, red) the respective radii are decreasing. This effect clearly influences the behavior of the lattice parameters and the shortest Si/*T* distance *d* of *R*Si_2_ and *R*_2_ *T*Si_3_ compounds. Another peculiarity of the lanthanides is that the metallic radii of Eu and Yb are 10% bigger than those of the other lanthanides. However, the ionic radii of Eu and Yb are comparable with those of the alkaline earth metals Ca, Sr, and Ba, which often leads to a common grouping of those elements (Ca with Yb as well as Sr and Ba, with Eu) (Evers *et al.*, 1980 ▸; Cardoso Gil *et al.*, 1999 ▸; Brutti *et al.*, 2006 ▸). These phenomena are caused by the stability of Eu and Yb in the +II oxidation state, due to half and completely filled *f* shells, respectively. A similar behavior does not occur for the actinides because the 5*f* shell experiences full shielding of the nuclear charge by the 4*f* shell. Because of the different energy levels of the respective orbitals, the electron configurations differ significantly between actinides and lanthanides.

Another consequence of the lanthanide contraction is that the radii of the 5d elements are very similar ot those of the 4d elements. Thus, these groups have similar sterical effects.

## Appendix B Discussion of the valence electron concentration

### b1. Determination and evaluation of the valence electron concentration

The determination of the amount of valence electrons, and thus of the valence electron concentration vec is challenging. In the following section, we present one approach that accords with a number of articles (Mayer & Felner, 1973*a* ▸,*b* ▸; Chevalier *et al.*, 1986 ▸). Here, we determine the number of valence electrons as the amount of those electrons that are capable to form chemical bonds. We set the valence electron number of elements with stable electronic configurations (according to the octet rule) to zero, *e.g.* noble gases as well as elements with completely filled *d* and *f* shells. For lanthanides the most typical oxidation state is dictated the valence electron number (*e.g.* two for Eu and Yb, three for other lanthanides). The completely filled *s* shells only count for elements of the main groups 1 to 4. And finally, unpaired electrons only count for *p* and *d* shells.

Another approach would be to use the amount of electrons in the outer shells for the determination of the vec, as done by (Cardoso Gil *et al.*, 1999 ▸; Chevalier *et al.*, 1984 ▸; von Schnering *et al.*, 1996 ▸).

### b2. Influence of the *T* element on vec and lattice parameters

In §4.5.4 we already discussed one approach to survey the finding of Mayer & Felner (1973*b* ▸) that a change in vec results in the weakening of some bonds and thus an elongation of the lattice parameters. For this part, we substitute the *T* element while keeping *R* constant. We chose *R* elements that form compounds with at least five different *T* elements, namely *R* = La, Ce, Eu, Dy, U or *R* = Th for ternary AlB_2_-like or ThSi_2_ structures, respectively (Fig. 22 ▸). Under the additional condition of similar radii, we analyzed the compounds according to the period of the *T* element. The majority of these series did not confirm the suggested vec rule. For most cases, the radius of the *T* element had a stronger influence, visible in a trough in the course of the lattice parameters at the elements of the groups Fe, Co, Ni.

### b3. Symmetry determined by vec?

Mayer & Felner (1973*b* ▸) analyzed *R*_2_ *T*Si_3_ compounds with *R* = Pr, Nd, Dy, Er and *T* = Fe, Co, Ni, Ag and found that those with a vec value below 3.4 are AlB_2_-like and those above are ThSi_2_-like. The more comprehensive data presented in Fig. 23 ▸ cannot confirm this limit. For example, the AlB_2_-like U compounds (Chevalier *et al.*, 1996 ▸; Pöttgen & Kaczorowski, 1993 ▸; Kaczorowski & Noël, 1993 ▸) have a vec of > 4.0 (above limit) and tetragonal Er_2_CuSi_3_ (Raman, 1967 ▸), Nd_2_AgSi_3_ (Mayer & Felner, 1973*b* ▸), and Ce_2_AuSi_3_ (Gordon *et al.*, 1997 ▸; Majumdar *et al.*, 2000 ▸) have a vec of 3.1667 (below limit). YSi_2_ has an even lower vec of 3.0 and is nevertheless tetragonal (Perri *et al.*, 1959*b* ▸,*a* ▸; Mayer *et al.*, 1962 ▸; 1959; Binder, 1960 ▸).

The respective boxplot (see Fig. 18 ▸) shows the distribution of vec for the different crystal lattices and reveals no correlation of lattice type and vec range. The only possible conclusion is the exclusive occurrence of orthorhombic GdSi_2_-type for vec = 4.67.

## Appendix C Influence of the Bader volume on the charge

The Bader analysis shows that the Bader charge directly depends on the Bader volume. The volume defines the amount of electron density ascribed to an atom.

Fig. 24 ▸ shows the values of the Bader charge *q*, the minimal distance *d*, and the Bader volume *V* determined for Nd_2_CuSi_3_ with structure type Er_2_RhSi_3_ (, No. 190). For a better comparison, the values are normalized to their maximum. The twelve different Si atoms form the *x*-axis. The division of the charge *q* by the volume *V* gives a much smoother trend than for the charge itself.

## Appendix D Further results of electronic correlations

In §4.5.4 we briefly discussed the thesis of Mayer *et al.* that a changing element would affect the *c* parameter in a stronger way than the *a* parameter (Mayer *et al.*, 1962 ▸; Mayer & Felner, 1973*a* ▸,*b* ▸). We already confirmed this thesis and would like to corroborate their findings with additional plots shown in Fig. 20 ▸.

Mayer *et al.* also concluded from their experiments on *R*_2_ *T*_*x*_Si_2−*x*_ compounds that a lower vec would lead to weaker and thus longer Si—*T* bonds compared to the covalent Si=Si bonds. As a result, the *c* parameter of ThSi_2_-like compounds would elongate and for AlB_2_-like compounds *a* would increase, whereas *c* would decrease (Mayer & Felner, 1973*a* ▸,*b* ▸). We have already shown with two examples that this theory cannot be applied to a series of *R*_2_ *T*Si_3_ compounds with changing *T* elements. Here, we want to provide the counter examples to Mayer’s theory with regard to ThSi_2_-like compounds in Fig. 25 ▸. For this case, the groups *R*Si_2_ with *R* ^*t*, 1^ = Ca, La, Eu, Yb and *R* ^*t*, 2^ = U, Np, Pu exist. Most of the compounds in Fig. 25 ▸(*a*) have an ionic character, as we discussed previously. Therefore, we present the ionic radii for the elements Ca, Eu, and Yb in addition to the metallic radii of La, Eu, and Yb. However, the trends of the lattice parameters do not confirm the theory for any of the choices of radii. Additionally, the series *R* ^*t*, 1^ exhibits decreasing *c* when the electron amount of *R* decreases, which also contradicts the theory.
